# Formation of Biphasic Hydroxylapatite-Beta Magnesium Tricalcium Phosphate in Heat Treated Salmonid Vertebrae

**DOI:** 10.1038/s41598-017-03737-2

**Published:** 2017-06-15

**Authors:** Don H. Butler, Ruth Shahack-Gross

**Affiliations:** Laboratory for Sedimentary Archaeology, Department of Maritime Civilizations, University of Haifa, 199 Abba Khoushy Ave, Haifa, 3498838 Israel

## Abstract

Ichthyoarchaeological evidence is uncommon at ancient hunter-gatherer sites from various regions and timeframes. This research contributes to the development of microarchaeological techniques useful for identifying fishing economies in situations where classifiable bones are unavailable. Specifically, traces of heat altered bone mineral in domestic hearths are expected to provide markers for discarded fish remains. We used a series of laboratory incineration experiments to characterize the mineralogy of burned salmonid vertebrae. Fourier transform infrared spectroscopy and x-ray diffraction distinguished the formation of beta magnesium tricalcium phosphate (βMgTCP) at temperatures as low as 600 °C. Bones from a sample of game mammals and birds did not form this phase at temperatures below 1,000 °C. We propose that this neoformed mineral can serve as a proxy for hunter-gatherer salmonid fishing when typical ichthyoarchaeological evidence is absent. Using Fourier transform infrared spectroscopy, it will be possible to rapidly and inexpensively determine the presence of βMgTCP in fragmentary burned bone remains associated with combustion features. The occurrence of βMgTCP in archaeological hearth features will offer a new means of further evaluating the temporal, geographic, and cultural scope of salmonid harvesting. We also acknowledge the value of biphasic hydroxylapatite-βMgTCP recovered from Atlantic salmon vertebrae as a bioceramic.

## Introduction

Salmonid resources have contributed to human progress for thousands of years. Today, salmonid aquaculture is our fastest growing food production industry with close to 3.5 million tonnes raised in 2014^1, 2^. Production growth supports socioeconomic development in several countries, while increased availability and competitive pricing is shifting consumer preferences toward farmed salmon, which is expected to divert stress from vulnerable wild stocks^2–5^. Oils, proteins, amino acids, and minerals extracted from farmed fish wastes also have diverse uses in food and medical engineering industries^6^. Fish bone, in particular, is a viable source of phosphate minerals useful for the fabrication of orthopedic bioceramics^7–10^.

Archaeological studies of salmonid harvesting societies have provided insight into the dynamics of socio-environmental relationships, resource management and economic diversification, power and circumscription, and movements throughout landscapes. Salmonids were fished by the end of the Middle Palaeolithic. Forty thousand year old remains discovered at Kudaro 3 cave in the South Ossetian Greater Caucasus evince the broadening of subsistence bases toward the end of the Pleistocene^11^. Ahead in time, and to the east, salmonids have been an economic pillar in Japan for thousands of years. During the 9^th^ century A.D., for example, communities processed fish locally and exported fillets to Kyoto to honour taxation responsibilities^12^. Across the Pacific, salmonid harvesting along the Northwest Coast of North America was at the centre of developments in sociocultural complexities among many hunter-gatherer groups, most prominently, the establishment of sedentary communities, the production of resource surpluses, and the organization of political structures^13^.

Aside from such examples, the importance of salmonid resources is unclear in many other places and timeframes. Documenting the significance of fishing among pioneering hunter-gatherers in the North American Arctic, for example, is challenged by an incomplete archaeological record^14^. Evidence for fishing is often lost. This owes to the consumption of waste bones by animals, the coarse sieving methods traditionally employed in Arctic archaeology, and perhaps most importantly, it owes to the poor survivorship of fish bones in many northern contexts^14–16^. Decomposition is controlled by interactions between the inherent characteristics of fish bone and the physical, chemical, and biological parameters of depositional environments. Fish bone can be easily degraded in well drained, microbially active, and acidic sediments because of its large organic component, loosely packed and poorly mineralized collagen bundles, and its porous structure^17–21^. Salmonid bones in specific can rapidly decompose because of their low mineral content and underdeveloped mineral-collagen interfacing^20, 21^. These characteristics offer greater access to bacterial enzymes, which accelerates collagen digestion and exposes more of the mineral surface area to weathering processes^21^.

Waste management strategies can provide insight into salmonid fishing activities when classifiable bone specimens are missing. Specifically, trash incineration was ubiquitous among past hunter-gatherers^22–26^. Domestic hearths were nodes of refuse disposal, and those built by salmonid fishers would have acted as sinks for burned fish bone. Therefore, we propose the use of heat altered bone mineral as a microarchaeological indicator for salmonid fishing. To our knowledge, mineralogical studies of bones are few in zooarchaeology in general and ichtyoarchaeology in particular. It is therefore of high importance to characterize the mineral properties of burned fish bones. Here, we report the results of laboratory heating experiments designed to characterize thermally induced changes in salmonid bone mineralogy. Fourier transform infrared spectroscopy (FTIR) and x-ray diffraction (XRD) were used to track alterations in the mineral component. We compared bone from three salmonid species with codfish, caribou, moose, duck, and ptarmigan bones to determine whether the salmonids present diagnostic mineralogical traits. Our results indicate that a newly formed phosphate mineral has potential as a marker for salmonid fishing where typical evidence is compromised. The occurrence of this mineral in hearths at hunter-gatherer archaeological sites will contribute to clarifying hunter-gatherer mobility strategies, economic diversification, and adaptations to frontier landscapes.

## Results

Heating experiments were undertaken on vertebrae from three individuals of Atlantic salmon (*Salmo salar*), steelhead (*Oncorhynchus mykiss irideus*), rainbow trout (*Oncorhynchus mykiss*), and Atlantic cod (*Gadus morhua*). One thoracic, one precaudal, and one caudal vertebrae were randomly selected from each individual (n = 36). Tibia from two individual caribou (*Rangifer tarandus groenlandicus*) and two individual moose (*Alces alces*), along with humeri from three individual ducks (*Anas sp*.) and a humerus from a ptarmigan (*Lagopus sp*.), were analysed for comparative purposes. Each sample was heat treated through a complete ramping sequence of 200, 400, 500, 600, 700, 800, and 1,000 °C for 1 h at each interval. Subsets of specimens were also exclusively fired at temperatures of 600, 700, 800, and 1,000 °C for varying durations.

**Figure 1:**
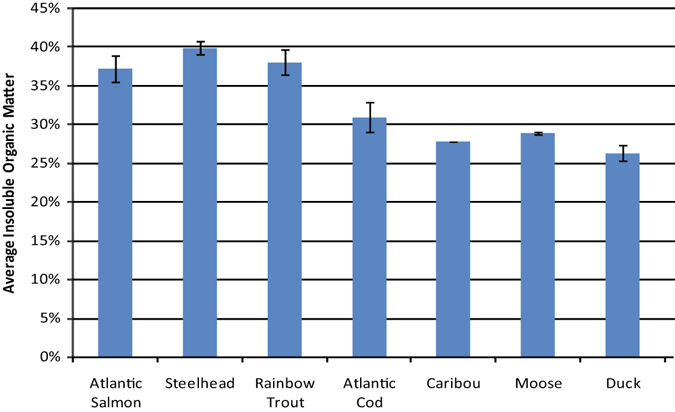
Content of Acid Insoluble Organic Matter Extracted from Unheated Air-Dried Salmonid, Codfish, Caribou, Moose, and Duck Bones. Results illustrate greater organic matter contents in salmonid specimens.

**Figure 2:**
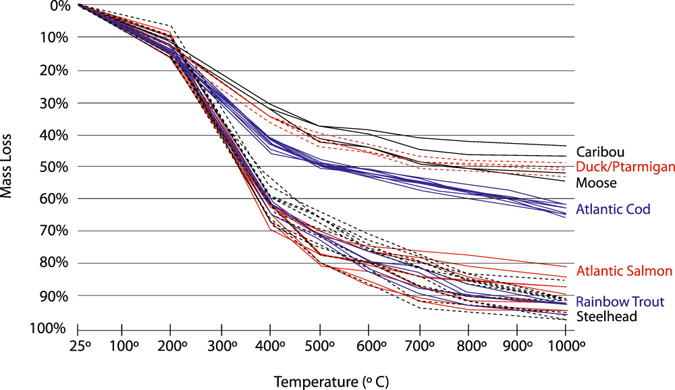
Mass Losses in Salmonid, Codfish, Caribou, Moose, Duck, and Ptarmigan Bones throughout the Heat Treatment Process. All species lost a similar amount of weight at 200 °C owing to evaporating H_2_O and melting fats; salmonid specimens show a sharp decrease at 400 °C resulting from greater losses of their larger organic matter fractions; caribou, moose, and duck contain the lowest amounts of organic matter and show similar mass loss trajectories.

Mass losses, FTIR, and XRD were used to characterize changes in the organic and mineral fractions of the bone. Weight losses displayed by unheated air-dried sub-samples after dissolution in 1N HCl showed that salmonid bones contained roughly 10% more acid insoluble organic matter than those of the other tested animals (Fig. 1). Differences in mass loss caused by thermal decomposition were also documented (Fig. 2). All of the salmonid vertebrae lost over 80% of their initial weights, while codfish vertebrae lost roughly 65%. Caribou, moose, duck, and ptarmigan bones lost between 44% and 55% of their initial weights. A sharp decrease in mass was observed in the salmonid samples heated at temperatures between 200 °C and 400 °C. They lost an average of 50% of their weight between these temperatures, while the other tested animals lost an average of 25% within this interval. Mass losses at 400 °C relate to the combustion of organic matter^27^. Greater losses recorded for salmonid bones in this range indicated higher organic matter contents. Mass losses above 400 °C primarily relate to the decomposition of bone mineral^27, 28^.

**Figure 3:**
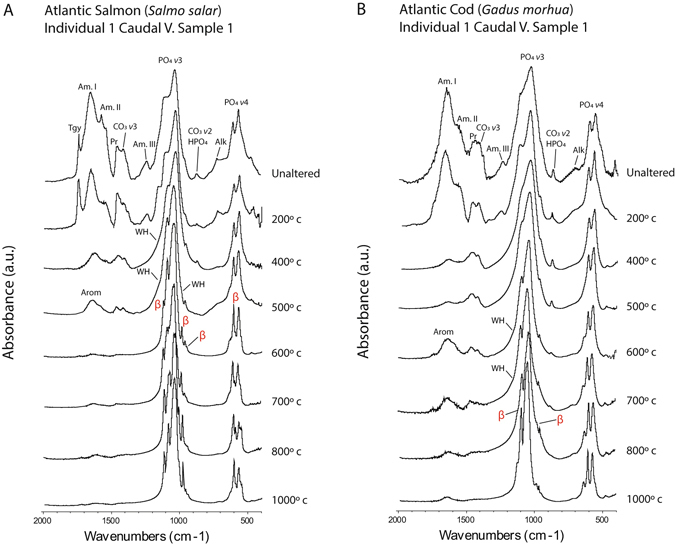
FTIR Spectra of Salmon and Codfish Bones Heat Treated through a Ramping Temperature Sequence between 200 °C and 1,000 °C. (**A**) Atlantic Salmon; note the loss of the HPO_4_/CO_3_ peak and appearance of strong βMgTCP peaks at 600 °C; (**B**) Atlantic Cod; the loss of the HPO_4_ / CO_3_ peak and appearance of weak βMgTCP peaks occurs at 800 °C; abbreviations: Tgy = triglyceride ester; Am = amide; Alk = alkene; Pr = proline; CO_3_ = carbonate; PO_4_ = phosphate; HPO_4_ = hydrogen phosphate; Arom = aromatic char compounds; WH = whitlockite; β = beta magnesium tricalcium phosphate.

**Figure 4:**
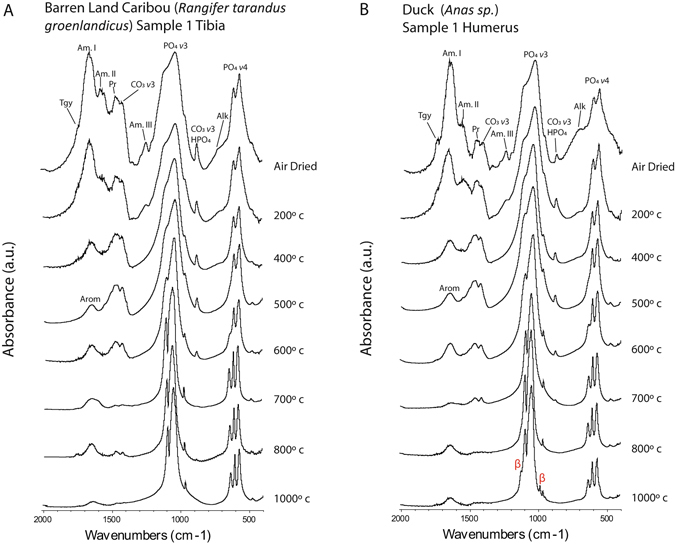
FTIR Spectra of Caribou and Duck Bones Heat Treated through a Ramping Temperature Sequence between 200 °C and 1,000 °C. (**A**) Caribou; βMgTCP does not form; (**B**) Duck; βMgTCP is only formed at high temperatures; abbreviations: Tgy = triglyceride ester; Am = amide; Alk = alkene; Pr = proline; CO_3_ = carbonate; PO_4_ = phosphate; HPO_4_ = hydrogen phosphate; Arom = aromatic char compounds; WH = whitlockite; β = beta magnesium tricalcium phosphate.

Losses of specific components were clarified by FTIR spectroscopy. Figures 3 and 4 display representative infrared spectra of Atlantic salmon, codfish, caribou, and duck bones obtained throughout the heating sequence. Supplementary Figures S2–S13 present the full dataset obtained from fish bones, while Supplementary Figures S14–S16 show all of the spectra obtained from caribou, moose, duck, and ptarmigan samples for comparison. Supplementary Table S1 lists the infrared bands tracked throughout the heating process. Peaks for fatty acids at 1,746 cm^−1^ and 722 cm^−1^ were significantly reduced between 400 °C and 500 °C in all of the tested samples^29^. Salmonid bones had more intense bands for these components in air-dried samples, indicating higher initial levels of fat. Amide peaks for collagen at 1,655 cm^−1^ (Amide I), 1,555 cm^−1^ (Amide II), and 1,240 cm^−1^ (Amide III) were reduced at 400 °C in all of the samples^30, 31^. The fish bones, however, displayed greater decreases in these components at 400 °C, suggesting a greater amount of organic combustion. This likely owes to the larger amounts of fat and insoluble organic matter in the salmonid samples. Bands in the 1,500–1,700 cm^−1^ region of the spectra for samples burned at temperatures above 500 °C are indicative of aromatic compounds of char residues from burned organic matter. This is expected in bone samples that have not been ashed completely (Figs 3 and 4)^32^.

Decarbonation and dehydration were also documented. However, tight proximity of the carbonate (CO_3_) *ν*3 peak at 1,420 cm^−1^ and the amino acid proline peak at 1,455 cm^−1^, as well as the CO_3_ *ν*2 peak at 875 cm^−1^ and the hydrogen phosphate (HPO_4_) peak at 880 cm^−1^, made it difficult to determine the exact timing of impacts on each component^27, 31–36^. Minor peak reductions in the proline and CO_3_ *ν*3 region were observed at 200 °C in the salmonid samples, followed by large reductions in the range of 400 °C to 500 °C. Both CO_3_ and HPO_4_ bands in the salmonid samples were lost between 600 °C and 700 °C, indicating excessive decarbonation and dehydration at lower temperatures than expected (Fig. 3A; Supplementary Figs S2–S10)^27, 34^. The CO_3_ and HPO_4_ bands were lost in caribou, moose, duck, and ptarmigan bones at 700 °C to 800 °C (Fig. 4; Supplementary Figs S14–S16).

**Figure 5:**
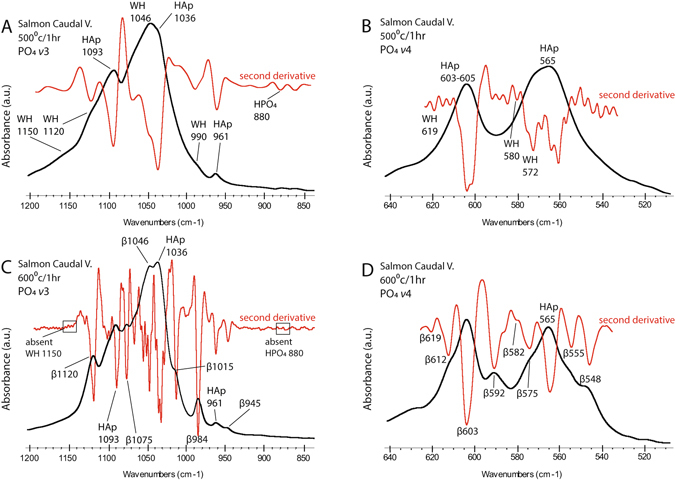
Magnified Representations of the Phosphate *v*3 and *v*4 FTIR Bands Demonstrating the Formation of Whitlockite and Beta Magnesium Tricalcium Phosphate Phases in Heat Treated Atlantic Salmon Vertebrae. (**A**) PO_4_ *v*3 band of a salmon caudal vertebra treated at 500 °C; diagnostic WH shoulders appear at 1,150 cm^–1^ and 990 cm^–1^; the presence of HPO_4_ demonstrated by the 880 cm^–1^ peak distinguishes WH from βMgTCP; (**B**) changes in the PO_4_ *v*4 band of a salmon caudal vertebra treated at 500 °C; (**C**) PO_4_ *v*3 band of a salmon caudal vertebra treated at 600 °C; loss of the diagnostic 1,150 cm^–1^ WH peak and the 880 cm^–1^ HPO_4_ peak and appearances of the 984 cm^–1^ and 945 cm^–1^ peaks signify a shift from WH to βMgTCP at 600 °C; (**D**) changes in the PO_4_ *v*4 band of a salmon caudal vertebra treated at 600 °C; abbreviations: HAp = hydroxylapatite; WH = whitlockite; β = beta magnesium tricalcium phosphate; HPO_4_ = hydrogen phosphate.

Infrared spectra of salmonids showed that the hydroxylapatite (HAp: Ca_10_(PO_4_)_6_(OH)_2_) mineral was significantly altered yet not entirely decomposed during the heating process. This was indicated by changes in the phosphate (PO_4_) *ν*3 at 1,035 cm^−1^, the PO_4_ *ν*1 at 961 cm^−1^, and the PO_4_ *ν*4 605/565 cm^−1^ doublet^27^. Specifically, we suspected the decomposition of a fraction of the HAp into a whitlockite (WH: Ca_9_Mg (HPO_4_)(PO_4_)_6_) phase based on the formation diagnostic shoulders on the PO_4_ *ν*3 band at 1,150 cm^−1^ and 990 cm^−1^ ^34, 35^. The 1,150 cm^−1^ shoulder was present in salmonid bone heated at 400 °C to 600 °C (Fig. 3A; Supplementary Figs 2–10). Both shoulders were well resolved in the second derivatives of samples burned at 500 °C (Fig. 5A). Changes in the PO_4_ *ν*4 band were also observed at 500 °C (Fig. 5B; Supplementary Fig. S17). Inconsistent formation was documented in codfish bone heated at temperatures between 600 °C and 700 °C (Fig. 3B; Supplementary Figs S11–S13). WH was not formed in the caribou, moose, duck, or ptarmigan samples (Fig. 4; Supplementary Figs S14–S16).

WH formation appears to be connected to the decalcification and decarbonation of HAp. Lower temperature alterations of organic matter and CO_3_ in the 1,400 cm^−1^ region can influence the B substitution site. The production of CO_2_ during carbonization creates vacancies in B sites that can cause Ca^2+^ deficiencies^33^. Decomposition of HAp into WH/βTCP is typical of bone with Ca^2+^ deficiencies^34^. Slight deficiencies (Ca/P ratios < 1.67) were confirmed by the XRD analyses (Supplementary Table S2)^8^. The WH phase appeared only in small amounts, and its presence was short-lived.

A phase change to tricalcium phosphate occurred in salmonid bones heated between 600 °C and 700 °C (Fig. 3A; Supplementary Figs S2–S10 and S17). Initial formation was marked by losses of the WH shoulder at 1,150 cm^−1^ and the CO_3_/HPO_4_ bands at 875–880 cm^−1^, along with the development of peaks at 1,120 cm^−1^ and 984 cm^−1^ (Fig. 5C)^35, 37, 38^. Peak positions in the PO_4_ *ν*3 and *v*4 regions suggested mineral lattice contraction caused by smaller ions occupying Ca^2+^ sites. This is characteristic of beta magnesium tricalcium phosphate (βMgTCP: MgCa_8_(PO_4_)_6_)^39, 40^. Lattice contraction owing to Mg^2+^ substitutions causes specific peaks to shift toward higher wavenumbers relative to those characterizing βTCP^40^. In our salmonid samples burned at 600 °C and above, peaks at 1,015 cm^−1^, 984 cm^−1^, and 945 cm^−1^ in the PO_4_ *ν*3 region and at 612 cm^−1^, 555 cm^−1^, and 548 cm^−1^ in the PO_4_ *ν*4 region were indicative of βMgTCP (Fig. 5C,D)^37, 40^. The mineral was also occasionally formed in codfish bones heated at 800 °C and 1,000 °C and in duck bones heated at 1,000 °C (Figs 3B and 4B; Supplementary Figs S11–S13 and S15). It was not formed in caribou, moose, or ptarmigan bone (Fig. 4A; Supplementary Figs S14 and S16).

**Figure 6:**
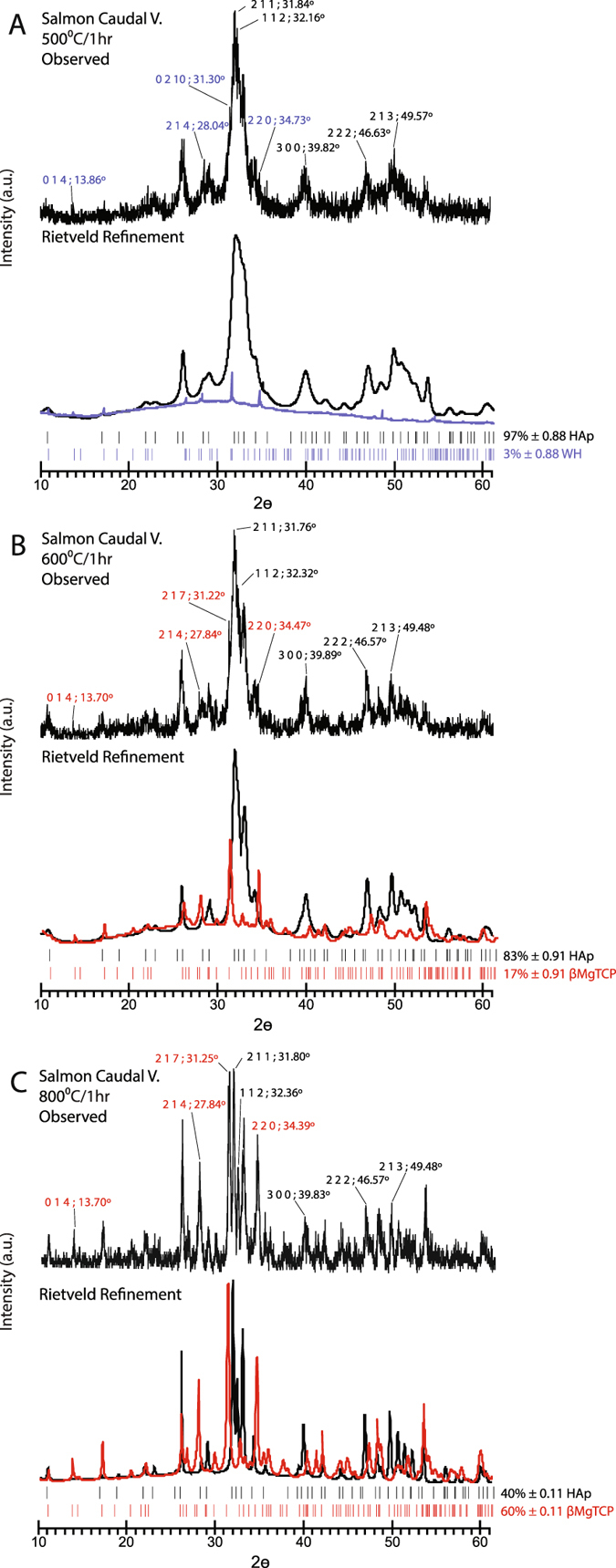
Observed XRD Diffractograms and Rietveld Refinements for Heat Treated Atlantic Salmon Bone. Each inset displays the observed diffractogram on top followed by refinement results on bottom; diagnostic reflectance planes and corresponding 2 θ angles for each phase are indicated in the observed diffractograms; separated phases are presented in the refinement sections; hydroxylapatite (HAp) is represented in black, whitlockite (WH) in blue, and beta magnesium tricalcium phosphate (βMgTCP) in red; (**A**) salmon caudal vertebra heat treated at 500 °C demonstrating diagnostic reflectance peaks for WH and HAp; (**B**) salmon caudal vertebra heat treated at 600 °C demonstrating diagnostic reflectance peaks for βMgTCP and HAp; (**C**) well developed biphasic HAp-βMgTCP in a salmon caudal vertebra after treatment at 800 °C.

XRD and Rietveld refinement confirmed the presence of WH and βMgTCP phases in heat treated salmonid bones (Fig. 6; Supplementary Fig. S18). Peak positions in our samples corresponded very well with JCPDS references for HAp (90432), WH (090169), and βMgTCP (130404)^40^. Both WH and βMgTCP are characterized by Mg^2+^ substitutions of Ca^2+^ and subsequent lattice contraction, which moves several diffraction peaks toward higher 2ϴ angles. WH also contains HPO_4_, causing additional contraction and further shifts than those presented by βTCP and βMgTCP. Previous research on this subject shows that the 0 2 1 0 reflectance plane for WH appears at a 2ϴ angle of 31.25°, while heated WH is distinguished by the emergence of this peak at 31.32°. The main reflection for βTCP is located at 31.02° and the 2 1 7 plane in βMgTCP reflects at 31.06°. HAp displays its most prominent peak representing the 2 1 1 reflectance plane at 31.80° ^34, 37, 38, 40^.

Atlantic salmon vertebrae treated at 400 °C contained a small amount of heat altered WH indicated by the position and intensity of the 0 2 1 0 peak at 31.36° (Supplementary Table S2). The heat altered WH concentration increased slightly at 500 °C. There was also a leftward shift of the 0 2 1 0 peak to 31.30° suggesting a relaxation of lattice contraction caused by the reduction of HPO_4_ (Fig. 6A). The loss of HPO_4_ was observed in the FTIR spectra (Fig. 5C). At 600 °C the 31.22° peak represented the 2 1 7 reflectance plane of βMgTCP (Fig. 6B). This peak shifted slightly to 31.25° at 800 °C (Fig. 6C). This is the same position as the 0 2 1 0 reflectance plane for WH. The mineral can not be classified as WH because of the absence of HPO_4_ in the structure. Moreover, if WH persisted at temperatures of 600 °C and beyond, we would expect the mineral to display its principal peak for the 0 2 1 0 reflectance plane at a 2ϴ angle of 31.32° or slightly higher^34^. Similar results were obtained for steelhead samples (Supplementary Fig. S18).

**Figure 7:**
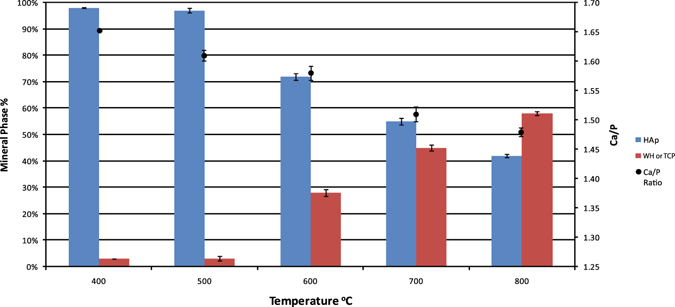
Mineral Phase Percentages in Heat Treated Atlantic Salmon Bone in Relation to the Ca/P Ratio of the Biphasic Mineral. Results demonstrate an inverse relationship between βMgTCP formation and the Ca/P ratio of the Biphasic Mineral.

Rietveld refinement enabled the calculation of several parameters (Supplementary Table S2). Ca/P ratios were lower than the HAp value of 1.67. An average Ca + Mg/P ratio of 1.50 for the neoformed βMgTCP corresponds well with previously published values^40^. Ca/P ratios for the composite decreased throughout the ramping burning sequence. This suggested that Ca^2+^ experienced gradual depletion. Transformation of a fraction of the HAp into WH and βMgTCP likely contributed to lowering the Ca/P ratio of the compound (Fig. 7; Supplementary Table S2)^41^. FTIR showed that Ca(OH)_2_ formed in salmonid bones heated above 600 °C, which may have also contributed to the diminishing Ca/P ratios observed in the HAp-βMgTCP composite (Supplementary Fig. S1). The Mg^2+^ present in the new mineral structure could have been sourced from the large organic component of the bone, yet organic matter was predominantly lost at 400 °C and completely lost at 600 °C (Fig. 3A; Supplementary Table S2; Supplementary Figs S2–S10). The production of βMgTCP continued to increase with heat treatment beyond 600 °C, suggesting the Mg^2+^ involved in its formation was sourced from the HAp mineral (Fig. 7).

Different ratios of HAp to βMgTCP were produced in Atlantic salmon vertebrae by controlling the firing temperature and duration of exposure (Supplementary Table S2). A HAp to βMgTCP ratio of 70–75:30–25% was produced at 600 °C after 2 h of heat treatment. A composite with a ratio of roughly 1:1 was formed at 700 °C and various exposure timings. The largest amounts of βMgTCP observed in the tested samples, upward of 60%, were produced at 800 °C (Fig. 6; Supplementary Fig. S19).

## Discussion

In summary, βMgTCP formation in salmonid bone involves the combustion of its larger organic component followed by the decarbonation, decalcification, and dehydration of HAp in the presence of Mg^2+^ ^35, 37–40^.The processes of HAp decomposition and new mineral formation documented in our salmonid bone samples are similar to those recorded for other animal bones^7, 8, 33, 42–44^. What differs in our results, however, is the lower formation temperatures observed for βMgTCP and the large amounts produced. Our findings have relevance in both archaeology and bioceramics engineering.

Previous experiments demonstrate that wood-fuelled hearths typically sustain temperatures of 500 °C to 600 °C^45–48^. Temperatures can spike to 800 °C, usually during the early stages of combustion, while temperatures of 1,000 °C are uncommon^48^. Archaeological research also provides evidence for hunter-gatherer hearths reaching temperatures between 500 °C and 700 °C^24, 49^. Firing conditions in wood-fuelled hearths would therefore be suitable for driving phase changes in salmonid bones, yet they would not typically reach temperatures high enough to produce dramatic transformations in other types of animal bones^24, 27, 36, 44, 48, 49^. Based on our results, it is not possible for βMgTCP to form within caribou, moose, duck, or ptarmigan bones – all typical game animals in Arctic environments – at temperatures below 1,000 °C. The formation of βMgTCP is specific to salmonid bones at temperatures as low as 600 °C, meaning its presence in combustion features at hunter-gatherer sites associated with salmonid migration routes can provide a marker for the disposal of salmonid bone wastes.

Burned fish bone, while often showing reasonable potential for preservation and serving as direct evidence for human agency, can be friable, crumbling into particles that may escape recovery during archaeological excavation, even when using the 1 mm sieve sizes recommended for maximizing the retrieval of fish bone^14, 23, 50, 51^. Despite their fragile nature, fragments are expected to be concentrated in the sediments of combustion features. We propose that burned fish bone fragments can preserve better than unheated fish bones owing to (a) a decrease in bone porosity caused by the onset of mineral crystal sintering that lowers the amount of mineral surface area directly exposed to chemical weathering processes, and (b) their occurrence in dense burned bone deposits may induce local alkaline conditions that will protect bone mineral from severe diagenesis, thereby preserving a fraction of the deposited materials^7, 20^.

Moreover, WH and βTCP have been discovered in archaeological contexts of considerable time depth. Both were preserved in Middle Stone Age deposits excavated at Sibudu Cave in South Africa^52^. From the same region and a similar time depth, WH nodules were identified in the sediments of Elands Bay Cave^53^. WH was also preserved in 400,000 year old sediments from Cueva del Ángel in Spain^54^, in a 17,000 year old layer at Carpenter’s Gap rock shelter in Australia^55^, and in sediments from 2,500 year old open-air ritual immolation sites in the Eastern Alps^56^. These examples indicate that WH and βTCP mineral species can preserve over long periods of time. Research in the field of bioceramics engineering further demonstrates that WH and βTCP are relatively stable phases. WH is stable at pH levels as low as 4.2^34^. HAp and βTCP have comparable solubilities at pHs between 4 and 5, while βMgTCP is less soluble than HAp at pHs lower than 6^35, 57^. These data suggest that βMgTCP sourced from burned salmonid bone should have the potential to preserve in archaeological contexts. Additionally, βTCP and βMgTCP have been reported as high temperature phases, meaning it is implausible that they will form in naturally decomposing bone^7, 27, 32^.

Where produced, preserved, and distinguished from secondary mineral assemblages, βMgTCP in hearth sediments can provide another line of proxy evidence for fishing activities where classifiable ichthyoarchaeological remains are absent. The identification of salmonid fishing in new cultural, temporal, and regional contexts will provide evidence useful for clarifying seasonal mobility, economic diversification, and adaptations to frontier landscape. For example, two recent studies contribute to documenting the importance of salmonid resources among the early Palaeoindian people moving through Alaska^14, 23^. Eleven thousand year old salmon remains were identified using aDNA, lipid, and stable isotope analyses. Some bones did yield viable aDNA, yet retrieval was reduced because of the burned state of several specimens. This, together with the high cost and low throughput of such analyses, resulted in a relatively small sample size. FTIR will provide a rapid and inexpensive microarchaeological tool for confirming the presence of heat altered salmonid bone mineral in hearth contexts from Palaeoindian sites in the region. Mineralogical investigations of hearth sediments will thusly contribute to resolving the spatiotemporal scope of salmon fishing among pioneering Palaeoindian groups in eastern Beringia, as well as cultures in other Arctic regions such as northwestern Europe.

Another archaeological implication drawn from our study concerns the use of heat altered bone to estimate the temperatures of ancient fires. Several experimental and archaeological studies have used the height of the CO_3_ *v*2 peak, the width at half maximum of the PO_4_ *v*3, the evolution of a hydroxyl group band in the PO_4_ *v*4 region, and the degree of splitting between the peaks comprising the PO_4_ *v*4 to determine firing temperatures^24, 27, 48, 49, 58^. Our burning experiments on fish, mammal, and bird bone show that changes the CO_3_ and PO_4_ absorbance regions are not uniform across different animals. This is most clearly demonstrated in the salmonid bone results. The low initial absorbencies for the CO_3_ *v*2, its loss at temperatures lower than expected, and the formation of βMgTCP indicates that previously proposed temperature estimation methods are not applicable to salmonid bones. This suggests that models for firing temperature estimation should be developed at the species level. Firing temperature estimation may only be possible after the animal species is identified and the thermal diagenesis pathway for their bone is experimentally defined, thereby calibrating the estimation method on a case by case basis.

The results of our experiments also contribute to a growing body of research that promotes bone incineration as a sustainable means of upcycling wastes generated by the aquaculture industry^6–10, 44^. As stated above, Atlantic salmon is one of the most abundantly farmed species. Processing to satisfy market demands produces close to two hundred thousand tonnes of heads and bones annually^59^. These bone wastes can provide a valuable source of PO_4_ minerals useful in many fields, among them the field of bioceramics. Our findings show that Atlantic salmon bones are a favourable candidate for the production of biphasic HAp-βMgTCP. Most importantly, exposure time experiments demonstrate that Atlantic salmon vertebrae can be used to easily produce a range of HAp-βMgTCP ratios. This straightforward means of controlling production ratios has potential applications in the fabrication of minerals for specific purposes. For example, a HAp-βMgTCP ratio of 70–75%:25–30% is optimal for bioceramic bone implants^8, 60^. This ratio can be efficiently and inexpensively produced by heat treating cleaned and air-dried Atlantic salmon vertebrae at 600 °C for 2 h.

The Ca/P ratio of the composite is 1.59, improving the prospective of using it as a bone implant material. Bioceramics with Ca/P ratios lower than 1 are unsuitable as implants because of their high solubilities^61^. The HAp component provides an osteoconductive scaffold for the production of new bone, while the βMgTCP fraction is highly resorbable^9^. This aids bone remodelling by saturating implant contexts with PO_4_^−3^ ions that accelerate new bone formation, thereby improving interfacing between introduced minerals and existing bone^7, 61–63^.

## Conclusion

The formation of βMgTCP in burned salmonid bone opens another potential way to bypass the difficulties of detecting archaeological evidence for ancient harvesting activities. Our results show that βMgTCP can form with comparatively low temperature heating, in turn suggesting that formation is possible in wood-fuelled hunter-gatherer hearths. This mineral has promise as an indicator for burned salmonid bone at ancient hunter-gatherer sites and thus for evaluating the antiquity of salmon harvesting, even where classifiable bone specimens are lacking due to incineration, poor preservation, or a combination thereof. This discovery is expected to generate new knowledge in future research concerning the development of subsistence practices, human-environment interactions, niche construction, and social behaviour. Additional experimental and archaeological research will focus on the diagenesis and preservation of the mineral. Bioceramics extraction from the waste bones of farmed fish also contributes to diversifying aquaculture sustainability by promoting refuse upcycling. The production of biphasic HAp-βMgTCP from Atlantic salmon waste bones may provide a natural and inexpensive complement to synthetic bioceramics.

## Methods

All fish and duck samples were tested in triplicate using different individuals. Caribou and moose were duplicated using different individuals. One ptarmigan was available at the time of analysis. Defleshing and cleaning were done under warm running water using a scalpel and nylon brush. Temperatures did not reach boiling point, ensuring that alteration prior to the heating experiments was not an issue. Samples were air-dried. Remaining tissue was removed using a scalpel^58^. All samples used in the experiments were similar in size, roughly the size a of fish vertebrae (~1 cm * 1 cm * 1 cm).

Organic matter content was determined using a sub-sample of each unheated air-dried bone. Acid insoluble fractions were extracted to determine the amount of organic matter present. Roughly 100 mg of crushed sample was dissolved in 5 ml of 1 N HCl at room temperature. The extraction time was 2 h. Extracts were washed with distilled water, centrifuged at 4,400 rpm for 2 min, and oven-dried at 50 °C. Mass loss after treatment indicated the amount of mineral that dissolved, and thus the amount of insoluble organic matter remaining^48^.

Unaltered air-dried bones were burned in an electric laboratory furnace (MRC, MSF 12/6). All samples were heated in open atmosphere through a complete sequence of 200, 400, 500, 600, 700, 800, and 1,000 °C for 1 h at each interval. The oven was preheated to the desired temperature before samples were introduced. Specimens were removed from the oven after each interval and weighed, sub-sampled, reweighed, and placed back into the preheated oven for treatment at the next temperature^58^. Sets of specimens were also fired exclusively at temperatures of 600, 700, 800, and 1,000 °C for varying durations. Samples were weighed at each stage before and after firing on an analytical balance (Sartorius, Entris) to 0.1 mg accuracy.

All of the samples were analysed using an FTIR spectrometer (Nicolet iS5, Thermo Scientific) to characterize changes in mineral and organic components throughout the heat treatment sequence. A roughly 1:20 ratio of sample to potassium bromide (KBr) was homogenized and ground using an agate mortar and pestle. The blend was evenly dispersed inside a steel die and pressed at 2,000 kg for 60 s. Spectra were averaged from 32 scans in the 4,000 to 400 cm^−1^ region at 4 cm^−1^ resolution^27^. Linear baseline correction was applied where necessary. We used the first difference second derivative function provided by Omnic software v. 9.3.30 to resolve changes in the PO_4_ *ν*3 and *ν*4 bands of HAp. This approach clarified the positions of shoulders and peaks indicative of neoformed minerals. It was important to confirm the positions of shoulders/peaks at 1,150 cm^−1^ and in the 990–980 cm^−1^ region to distinguish WH (1,150, 990 cm^−1^) and βMgTCP (984 cm^−1^). Analyses were also used to confirm that the diagnostic 1150 cm^−1^ WH peak was not hidden by the dominant PO_4_ *v*4 band at temperatures of 600 °C and greater^34^.

Fifteen Atlantic salmon and four steelhead samples were analysed by XRD to confirm and quantify mineral phases. Atlantic salmon samples treated at 600 °C for 2 h and 800 °C for 1 h were tested in triplicate to ensure reproducibility. Samples were ground into powders with an agate mortar and pestle. These were evenly dispersed across the sample mount and compacted with a spatula. Diffractograms were collected using a Rigaku Miniflex 600. The instrument ran at 30 kV and 10 mA. It was equipped with a Cu x-ray tube (λ = 1.5418 Å), vertical goniometer, Kβ foil filter, and an SC-70 detector. The incident slit was 1.250° and the length of the limiting slit was 10 mm. Receiving slits 1 and 2 were 1.250° and 0.3 mm respectively. Incident and receiving soller slits were both 2.5°. Data was collected in the angular 2ϴ range of 10 to 60° with a step width of 0.02°. The scan speed was 4°/min.

Mineral phases were characterized through Rietveld refinement using Profex software v. 3.9.2. The procedure fits a model of hypothesized mineral phases to the observed diffractogram using a non-linear least squares approach. Model quality was assessed using the chi-squared goodness-of-fit statistic. Values were close to 1, indicating good correspondences between observed and refined diffractograms. Weight percentages of Ca, Mg, and P were automatically calculated by Profex using crystallographic indicators of site occupancies and the atomic weights of each site’s species. These were converted into molar masses to calculate element molar ratios. The software estimated standard deviations from assessments of noise amplitude^64^.

### Data Availability

All data generated or analysed during this study are included in this published article (and its Supplementary Information files).

### Ethics Statement

Fish used in this study were legally purchased from fish markets in Israel, Canada, and Mexico. One Atlantic salmon and one rainbow trout, along with the caribou, moose, and bird specimens, were sourced from Canadian fishers/hunters participating in licensed subsistence fishing/hunting. Permits were not required to possess the animal specimens. Animals were not live upon acquisition. No animals used in the project were sacrificed for research purposes. No animal used in the study is a threatened, endangered, or controversial species.

## Electronic supplementary material

Supplementary Figures
