# Supplementary material for: Formation of Biphasic Hydroxylapatite-Beta Magnesium Tricalcium Phosphate in Heat Treated Salmonid Vertebrae
Source: Sci Rep. 2017 Jun 15;7:3610. doi: 10.1038/s41598-017-03737-2 (PMC5472584; doi:10.1038/s41598-017-03737-2)

**Formation of Biphasic Hydroxylapatite-Beta Magnesium Tricalcium Phosphate in Heat Treated Salmonid Vertebrae**

Don H. Butler[1*] and Ruth Shahack-Gross[1*]

[1]Laboratory for Sedimentary Archaeology, Department of Maritime Civilizations, University of Haifa, 199 Abba Khoushy Ave, Haifa, 3498838, Israel

[*]Corresponding Authors: dhbutler@ucalgary.ca; rgross@univ.haifa.ac.il

This Supplementary file includes:
Supplementary Tables S1-S2
Supplementary Figs. S1-S19

## Supplementary Table 1: Peak Assignments used in the FITR Analyses

| Component | Wavenumber ($cm^{-1}$) | Group | Vibrational Mode | References |
|---|---|---|---|---|
| **Collagen** | | | | |
| | 1655 | Amide I | C=O stretch | Mao et al., 2016; Mary et al., 2009 |
| | 1555 | Amide II | C-N stretch<br>N-H bend | Mao et al., 2016; Mary et al., 2009 |
| | 1240 | Amide III | C-N stretch<br>N-H bend | Mao et al., 2016; Mary et al., 2009 |
| **Amino Acid** | | | | |
| | 1453 | Proline | $CH_2$ bend | DeNiro and Weiner, 1988; Mary et al., 2009 |
| **Fatty Acid** | | | | |
| | 1746 | Triglyceride | C=O stretch | Pu et al., 2014 |
| | 722 | Alkene | =C-H bend | Pu et al., 2014 |
| **Carbonate** | | | | |
| | 1420 | $CO_3$ ν3 | C-O stretch | Stathopoulou et al., 2008; Weiner, 2010 |
| | 875 | $CO_3$ ν2 | C-O bend | Stathopoulou et al., 2008; Weiner, 2010 |
| **Hydroxylapatite** | | | | |
| | 1035 | $PO_4$ ν3 | P-O bend | Stathopoulou et al., 2008; Weiner, 2010 |
| | 966 | $PO_4$ ν1 | P-O stretch | Stathopoulou et al., 2008; Weiner, 2010 |
| | 605-565 | $PO_4$ ν4 | P-O bend | Stathopoulou et al., 2008; Weiner, 2010 |
| | 480 | $PO_4$ ν2 | P-O bend | Stathopoulou et al., 2008; Weiner, 2010 |
| | 630 | OH | O-H libration | Blitz and Pellegrino, 1983; Goto and Sasaki, 2014; Reidsma et al., 2016 |
| **Hydrogen Phosphate** | | | | |
| | 880 | $HPO_4$ | P-O-H stretch | Jang et al., 2014; Li et al., 2009; Reidsma et al., 2016 |
| **Aromatic Compounds** | | | | |
| | 1500-1700 | Aromatics | C=C bend | Reidsma et al., 2016 |
| **Beta Magnesium Tricalcium Phosphate / Whitlockite** | | | | |
| | 1120 | $PO_4$ ν3 | P-O bend | Li et al., 2009; Stipniece et al., 2014 |
| | 1105 | $PO_4$ ν3 | P-O bend | Stipniece et al., 2014 |
| | 1097 | $PO_4$ ν3 | P-O bend | Li et al., 2009 |
| | 1088 | $PO_4$ ν3 | P-O bend | Li et al., 2009 |
| | 1075 | $PO_4$ ν3 | P-O bend | Li et al., 2009 |
| | 1046 | $PO_4$ ν3 | P-O bend | Li et al., 2009 |
| | 1042 | $PO_4$ ν3 | P-O bend | Li et al., 2009 |
| | 1036 | $PO_4$ ν3 | P-O bend | Stipniece et al., 2014 |
| | 1015 | $PO_4$ ν3 | P-O bend | Li et al., 2009 |
| | 998 | $PO_4$ ν3 | P-O bend | Li et al., 2009 |
| | 985 | $PO_4$ ν3 | P-O bend | Li et al., 2009 |
| | 970-974 | $PO_4$ ν3 | P-O bend | Li et al., 2009; Stipniece et al., 2014 |
| | 945 | $PO_4$ ν1 | P-O stretch | Stipniece et al., 2014 |
| | 619 | $PO_4$ ν4 | P-O bend | Li et al., 2009 |
| | 612 | $PO_4$ ν4 | P-O bend | Li et al., 2009 |
| | 592-594 | $PO_4$ ν4 | P-O bend | Li et al., 2009 |
| | 582 | $PO_4$ ν4 | P-O bend | Li et al., 2009 |
| | 565 | $PO_4$ ν4 | P-O bend | Li et al., 2009 |
| | 554-558 | $PO_4$ ν4 | P-O bend | Li et al., 2009; Stipniece et al., 2014 |
| | 603 | $PO_4$ ν4 | P-O bend | Li et al., 2009; Stipniece et al., 2014 |

| | | | |
|---|---|---|---|
| 548 | $PO_4$ ν4 | P-O bend | Li et al., 2009 |
| 990 WH | $PO_4$ ν3 | P-O bend | Li et al., 2009 |
| 1150 WH | $PO_4$ ν3 | P-O bend | Li et al., 2009 |

## Supplementary Table 2: Mineralogical and Elemental Data Obtained from XRD Rietveld Refinement Analyses

| | **HAp (wt. %)** | **βMgTCP (wt. %)** | **WH (wt. %)** | **Ca/P HAp** | **Ca/P WH or βMgTCP** | **Ca/P Biphasic Composite** | **Ca+Mg/P βMgTCP** | **Ca+Mg/P Biphasic Composite** | **Mg in WH or βMgTCP (wt. %)** |
|---|---|---|---|---|---|---|---|---|---|
| **Salmon** | | | | | | | | | |
| 400 ºC 1 h | 98%±0.1 | - | 2%±0.1 | 1.66 | 1.33 | 1.66 | - | 1.67 | 0.05% |
| 500 ºC 1 h | 97%±0.88 | - | 3%±0.88 | 1.66 | 1.38 | 1.61 | - | 1.63 | 0.1% |
| 600 ºC 1 h | 83%±0.91 | 17%±0.91 | - | 1.66 | 1.40 | 1.58 | 1.53 | 1.61 | 0.64% |
| 600 ºC 2 h | 75%±1.6 | 25%±1.6 | - | 1.66 | 1.40 | 1.59 | 1.50 | 1.62 | 0.98% |
| 600 ºC 2 h* | 70%±1.1 | 30±1.1 | - | 1.68 | 1.39 | 1.56 | 1.51 | 1.61 | 1.24% |
| 600 ºC 2 h* | 77%±0.8 | 23%±0.8 | - | 1.67 | 1.36 | 1.60 | 1.46 | 1.63 | 0.87% |
| 600 ºC 4 h | 64%±1.7 | 36%±1.7 | - | 1.68 | 1.37 | 1.56 | 1.49 | 1.60 | 1.40% |
| 700 ºC 1 h | 56%±0.11 | 44%±0.11 | - | 1.69 | 1.36 | 1.52 | 1.50 | 1.58 | 1.71% |
| 700 ºC 2 h | 56%±1.4 | 44%±1.4 | - | 1.66 | 1.37 | 1.48 | 1.49 | 1.57 | 2.19% |
| 700 ºC 4 h | 52%±1.1 | 48%±1.1 | - | 1.67 | 1.38 | 1.52 | 1.52 | 1.59 | 1.87% |
| 800 ºC 15 min | 43%±0.97 | 57%±0.97 | - | 1.68 | 1.38 | 1.48 | 1.50 | 1.56 | 2.24% |
| 800 ºC 30 min | 52%±0.96 | 48%±0.96 | - | 1.68 | 1.37 | 1.48 | 1.51 | 1.58 | 1.86% |
| 800 ºC 1 h | 40%±0.11 | 60%±0.11 | - | 1.66 | 1.39 | 1.48 | 1.52 | 1.56 | 2.32% |
| 800 ºC 1 h* | 42%±0.69 | 58%±0.69 | - | 1.67 | 1.40 | 1.48 | 1.50 | 1.56 | 2.24% |
| 800 ºC 1 h* | 44%±0.87 | 56%±0.87 | - | 1.66 | 1.38 | 1.48 | 1.52 | 1.56 | 2.22% |
| **Steelhead** | | | | | | | | | |
| 400 ºC 1 h | 98%±0.4 | - | 2±0.4 | 1.66 | 1.31 | 1.66 | - | 1.67 | 0.05% |
| 500 ºC 1 h | 91%±1.4 | - | 9%±1.4 | 1.68 | 1.32 | 1.59 | - | 1.61 | 0.31% |
| 600 ºC 1 h | 82%±0.9 | - | 18%±0.9 | 1.68 | 1.30 | 1.63 | - | 1.64 | 0.65% |
| 800 ºC 1 h | 45%±0.08 | 55%±0.08 | - | 1.68 | 1.37 | 1.48 | 1.50 | 1.54 | 2.16% |

**Supplementary Figure 1: Atlantic Salmon Individual 1 Caudal Vertebra FTIR Spectra of the 3,700 $cm^{-1}$ to 3,000 $cm^{-1}$ Region Illustrating the Formation of $Ca(OH)_2$ at 600 $^oC$, 700 $^oC$, 800 $^oC$, and 1,000 $^oC$.**

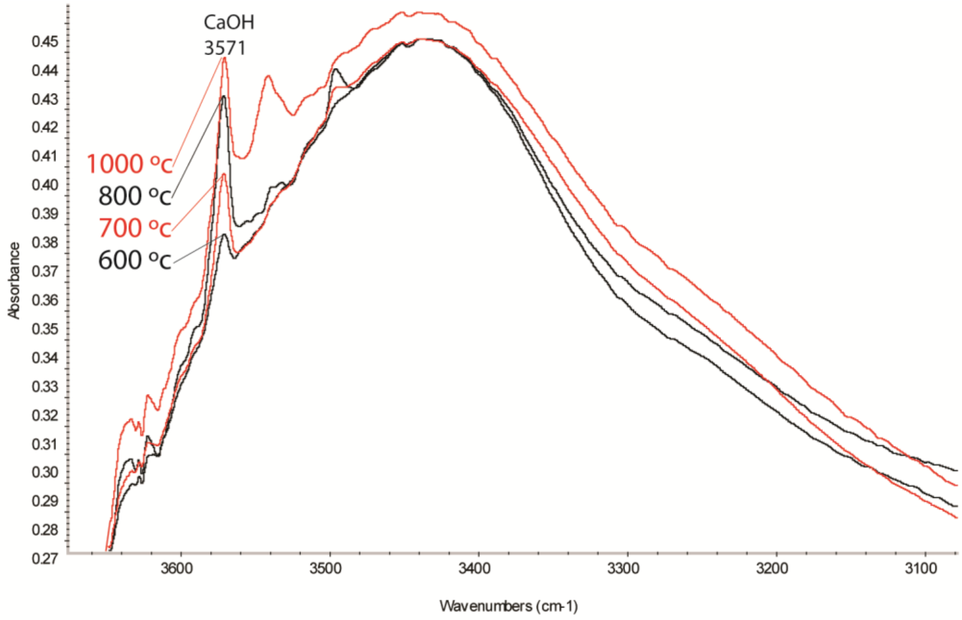

**Supplementary Figure 2: Atlantic Salmon Individual 1 Vertebrae FTIR Replications (Fingerprint Region).** Abbreviations for diagnostic peaks: Tgy = triglyceride ester; Am = amide; Alk = alkene; Pr = proline; $CO_3$ = carbonate; $PO_4$ = phosphate; $HPO_4$ = hydrogen phosphate; Arom = aromatic char compounds; WH = whitlockite; β = beta magnesium tricalcium phosphate.

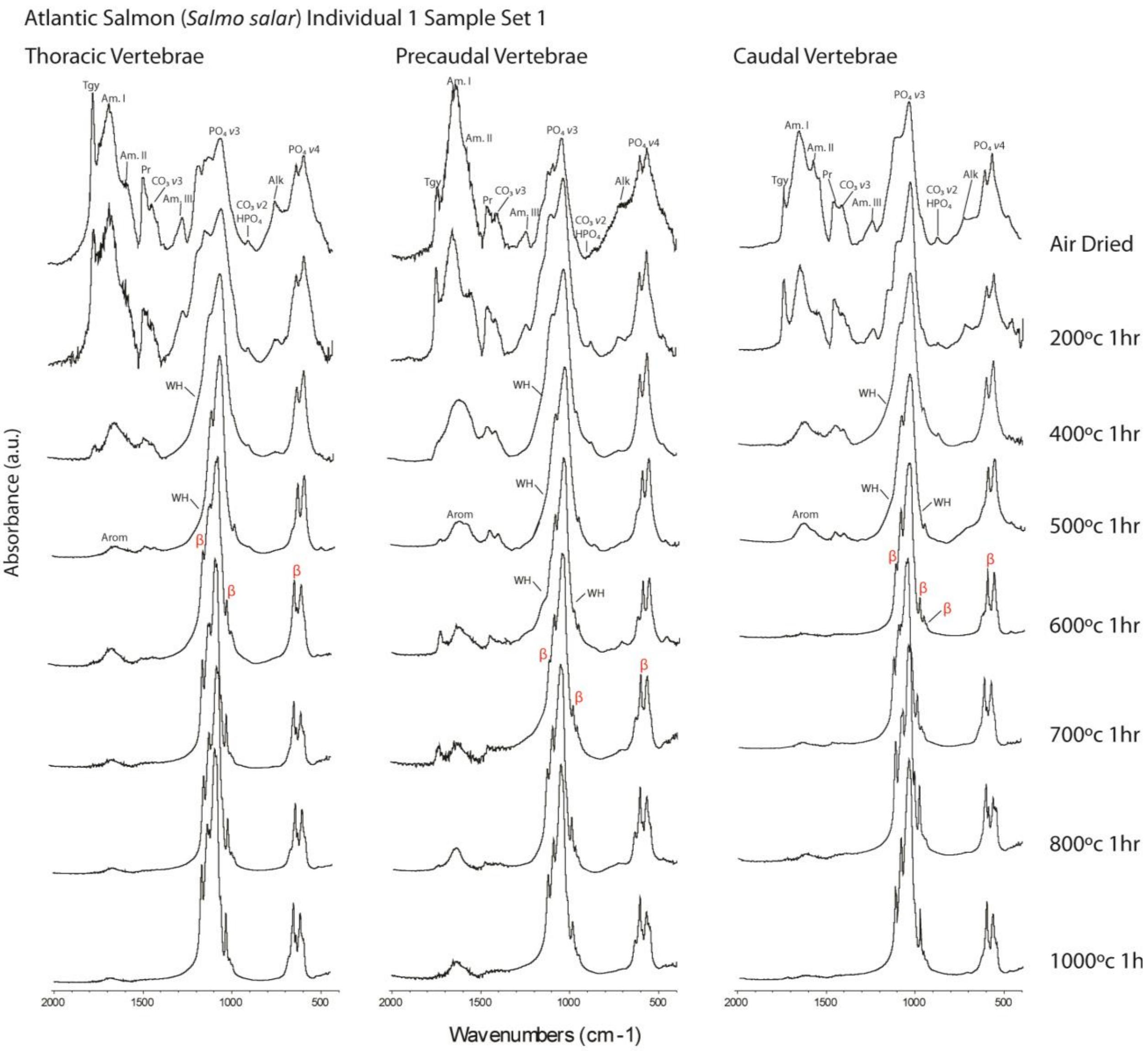

**Supplementary Figure 3: Atlantic Salmon Individual 2 Vertebrae FTIR Replications (Fingerprint Region).** Abbreviations for diagnostic peaks: Tgy = triglyceride ester; Am = amide; Alk = alkene; Pr = proline; $CO_3$ = carbonate; $PO_4$ = phosphate; $HPO_4$ = hydrogen phosphate; Arom = aromatic char compounds; WH = whitlockite; β = beta magnesium tricalcium phosphate.

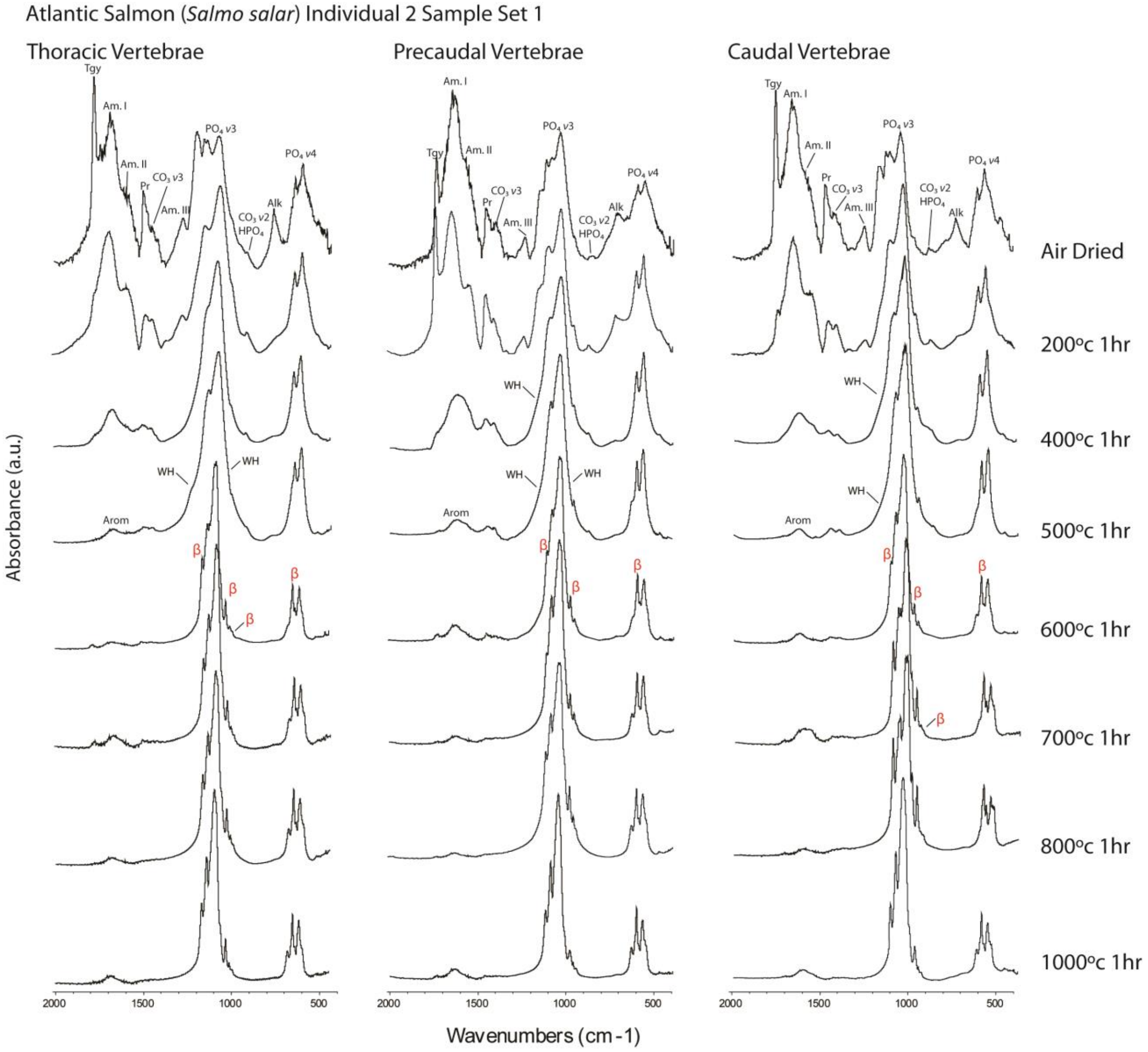

**Supplementary Figure 4: Atlantic Salmon Individual 3 Vertebrae FTIR Replications (Fingerprint Region).** Abbreviations for diagnostic peaks: Tgy = triglyceride ester; Am = amide; Alk = alkene; Pr = proline; $CO_3$ = carbonate; $PO_4$ = phosphate; $HPO_4$ = hydrogen phosphate; Arom = aromatic char compounds; WH = whitlockite; β = beta magnesium tricalcium phosphate.

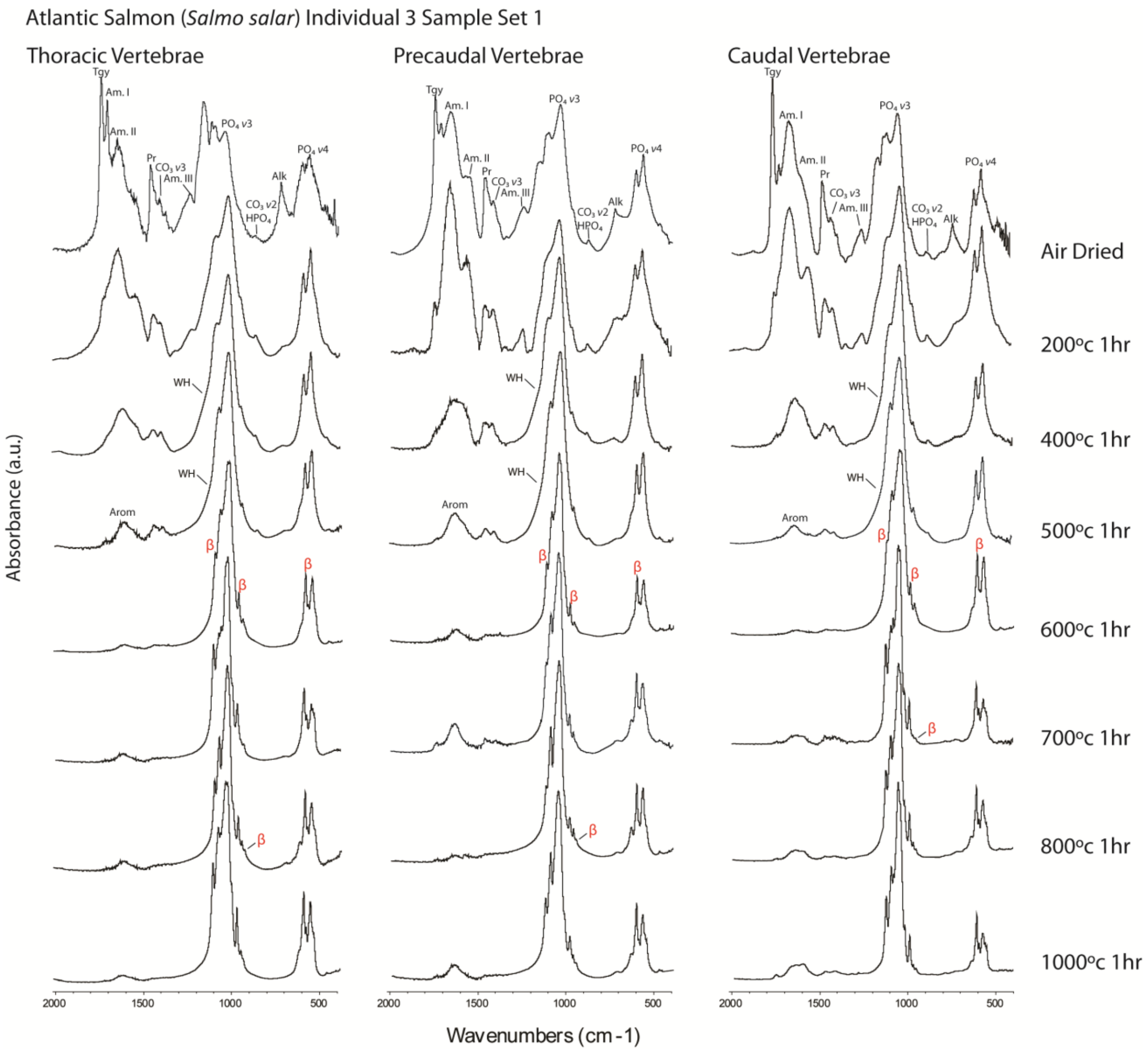

**Supplementary Figure 5: Steelhead Individual 1 Vertebrae FTIR Replications (Fingerprint Region).** Abbreviations for diagnostic peaks: Tgy = triglyceride ester; Am = amide; Alk = alkene; Pr = proline; $CO_3$ = carbonate; $PO_4$ = phosphate; $HPO_4$ = hydrogen phosphate; Arom = aromatic char compounds; WH = whitlockite; β = beta magnesium tricalcium phosphate.

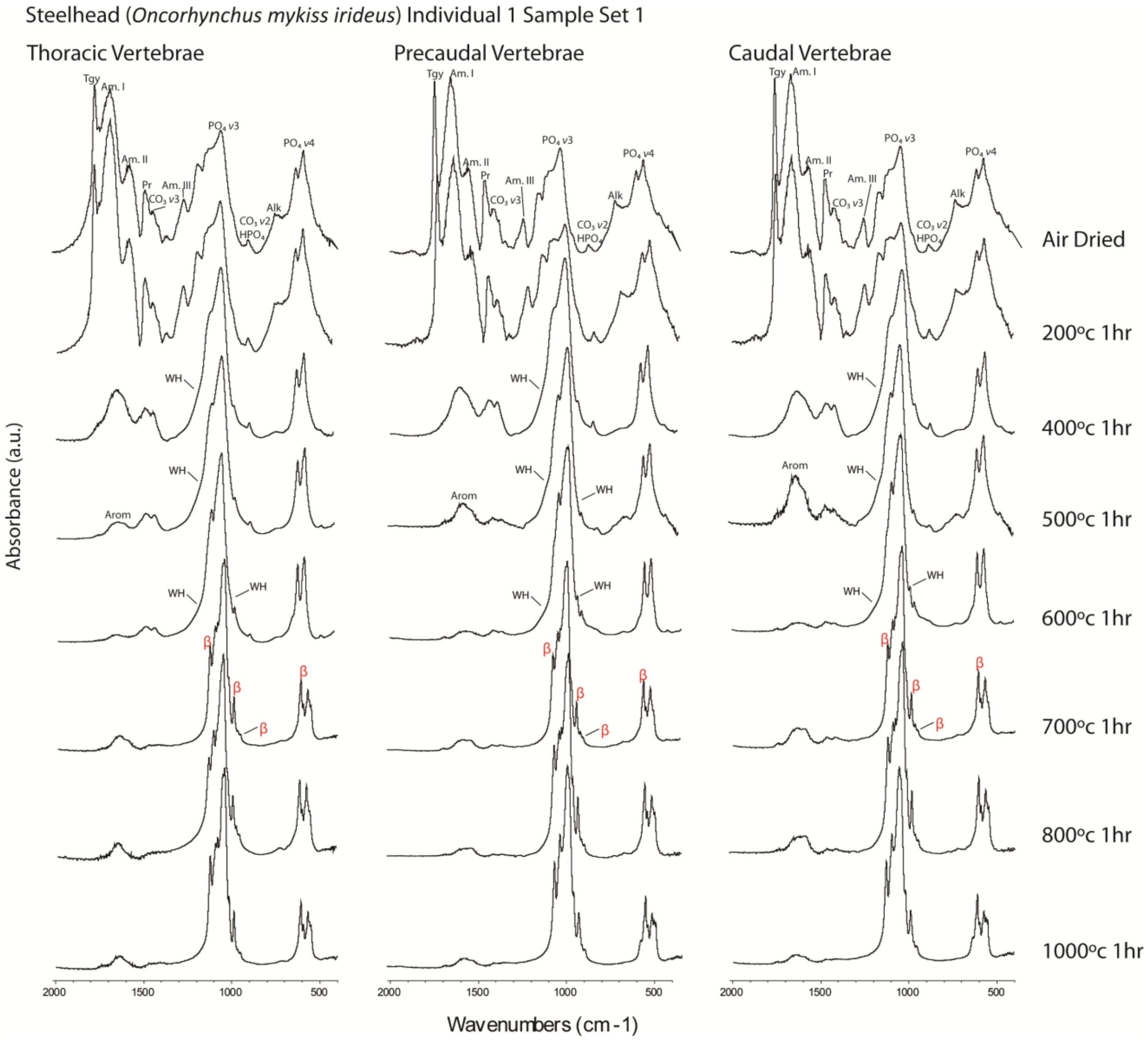

**Supplementary Figure 6: Steelhead Individual 2 Vertebrae FTIR Replications (Fingerprint Region).** Abbreviations for diagnostic peaks: Tgy = triglyceride ester; Am = amide; Alk = alkene; Pr = proline; $CO_3$ = carbonate; $PO_4$ = phosphate; $HPO_4$ = hydrogen phosphate; Arom = aromatic char compounds; WH = whitlockite; β = beta magnesium tricalcium phosphate.

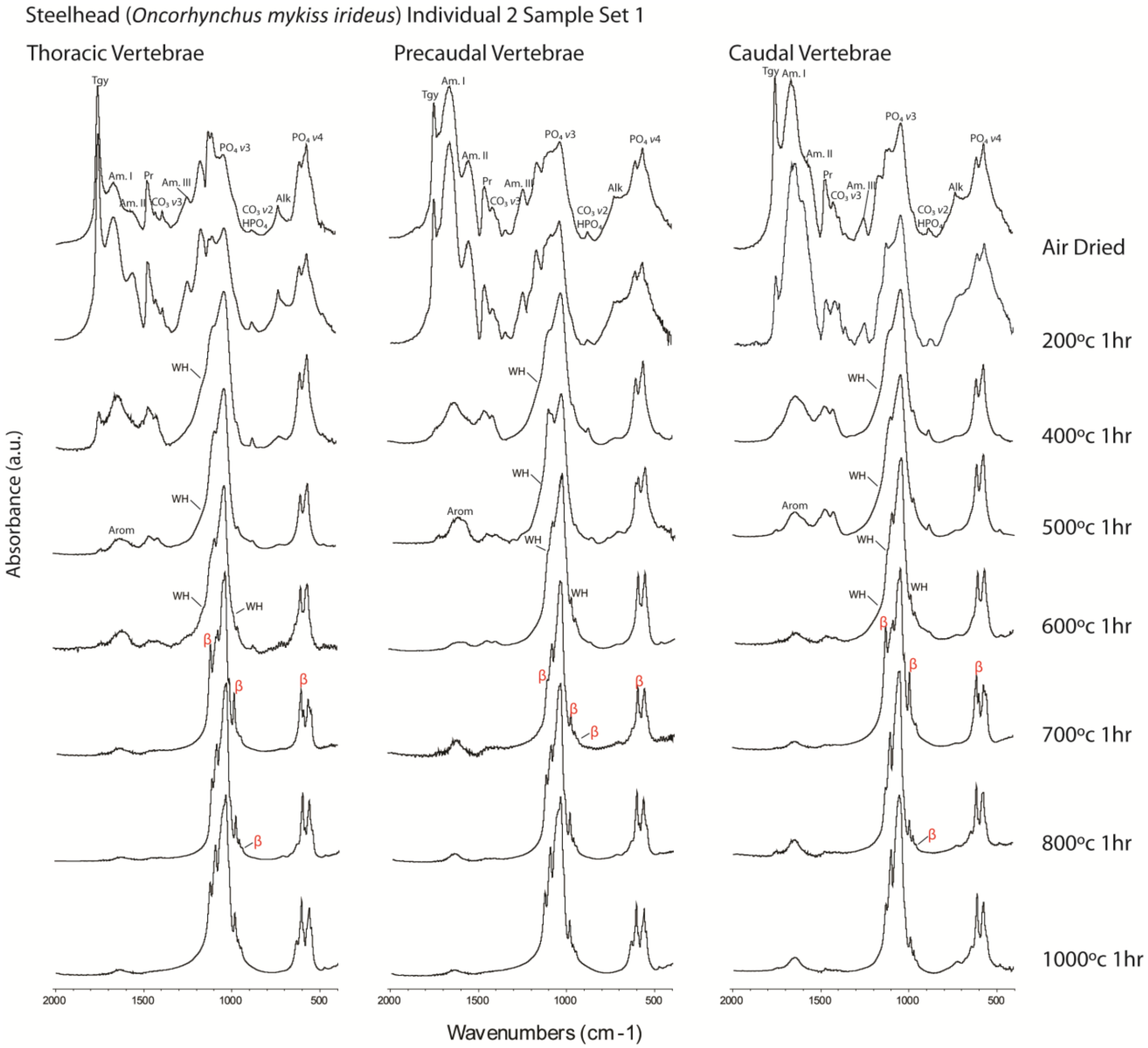

**Supplementary Figure 7: Steelhead Individual 3 Vertebrae FTIR Replications (Fingerprint Region).** Abbreviations for diagnostic peaks: Tgy = triglyceride ester; Am = amide; Alk = alkene; Pr = proline; $CO_3$ = carbonate; $PO_4$ = phosphate; $HPO_4$ = hydrogen phosphate; Arom = aromatic char compounds; WH = whitlockite; β = beta magnesium tricalcium phosphate.

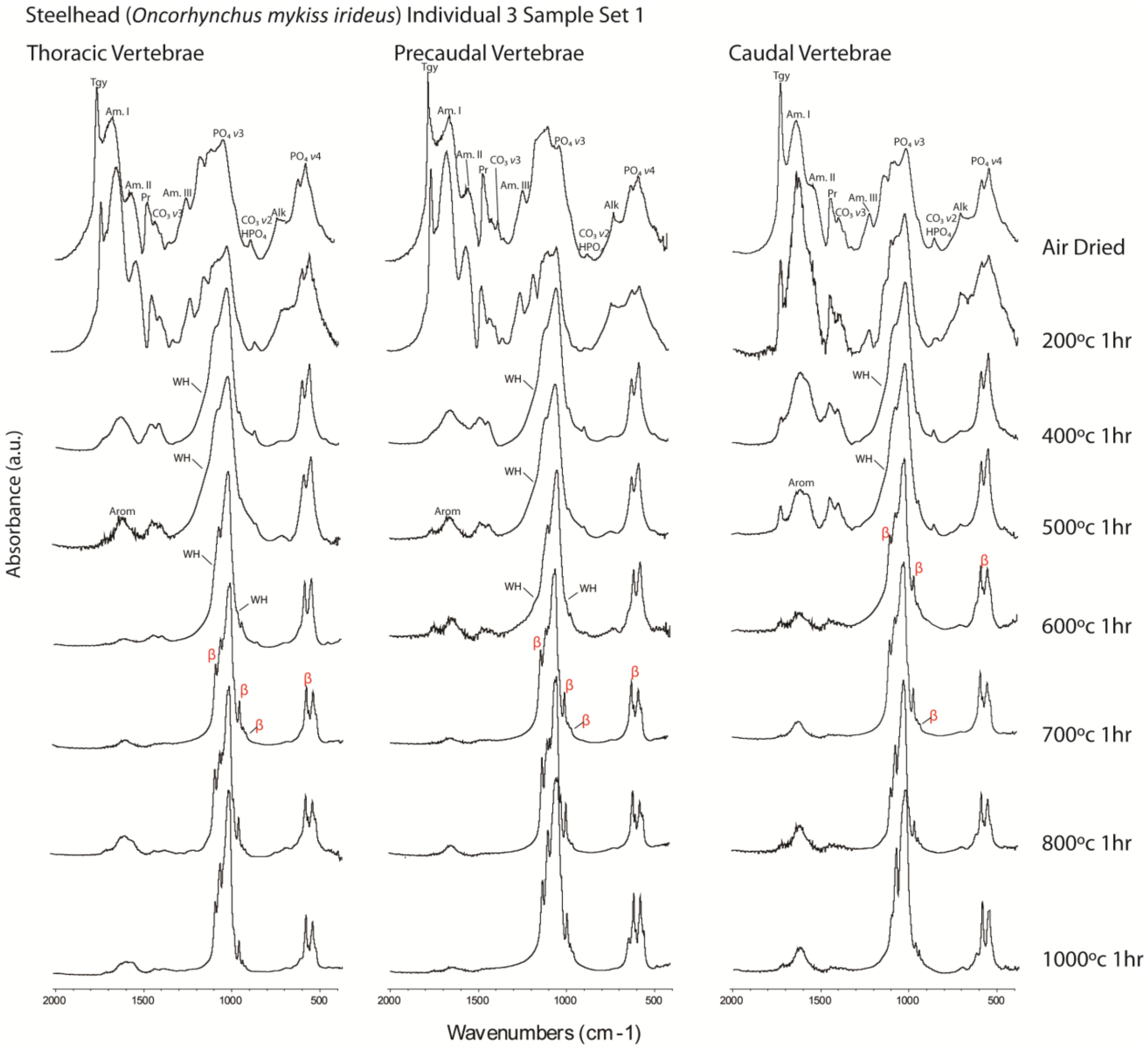

**Supplementary Figure 8: Rainbow Trout Individual 1 Vertebrae FTIR Replications (Fingerprint Region).** Abbreviations for diagnostic peaks: Tgy = triglyceride ester; Am = amide; Alk = alkene; Pr = proline; $CO_3$ = carbonate; $PO_4$ = phosphate; $HPO_4$ = hydrogen phosphate; Arom = aromatic char compounds; WH = whitlockite; β = beta magnesium tricalcium phosphate.

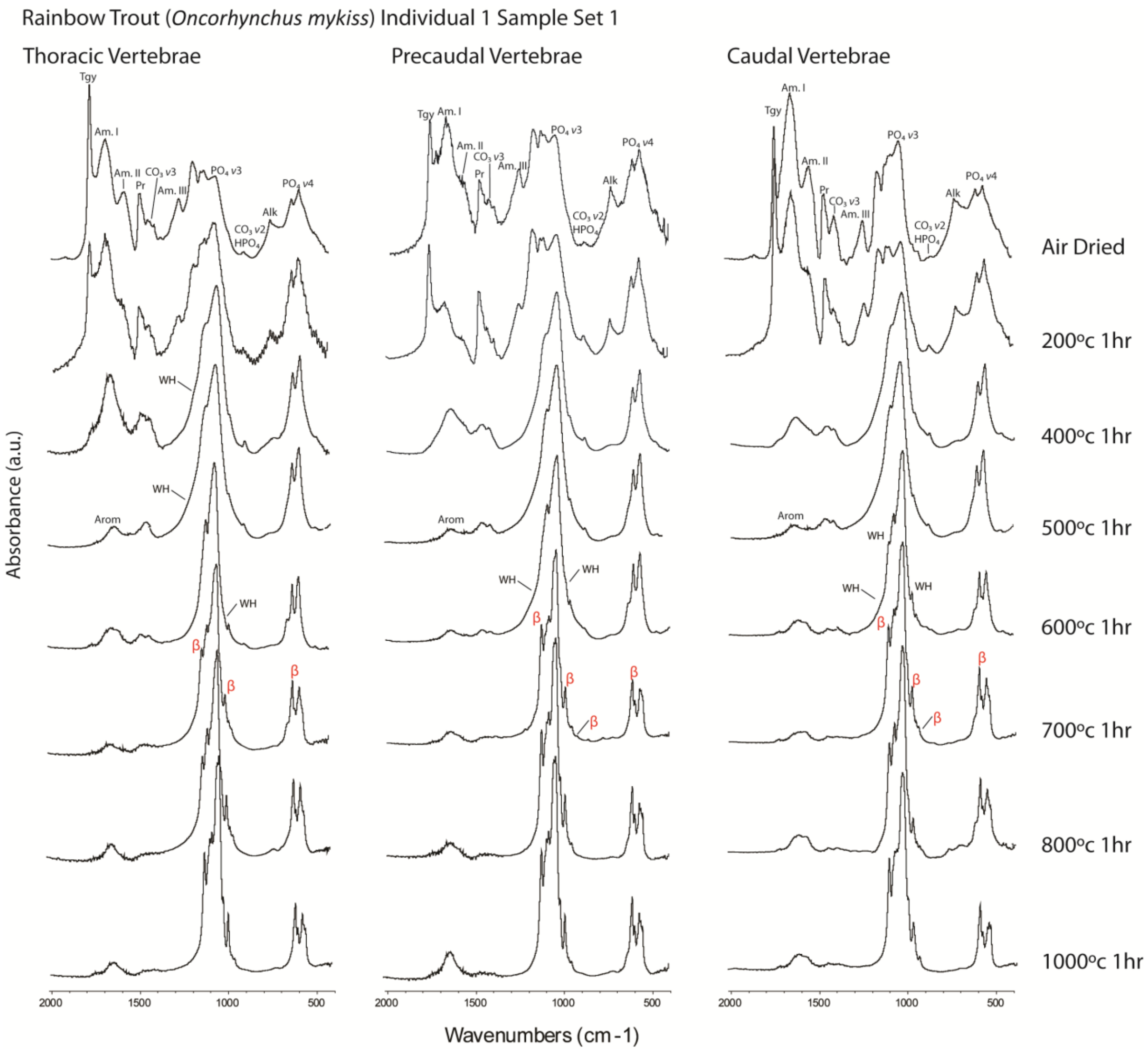

**Supplementary Figure 9: Rainbow Trout Individual 2 Vertebrae FTIR Replications (Fingerprint Region).** Abbreviations for diagnostic peaks: Tgy = triglyceride ester; Am = amide; Alk = alkene; Pr = proline; $CO_3$ = carbonate; $PO_4$ = phosphate; $HPO_4$ = hydrogen phosphate; Arom = aromatic char compounds; WH = whitlockite; β = beta magnesium tricalcium phosphate.

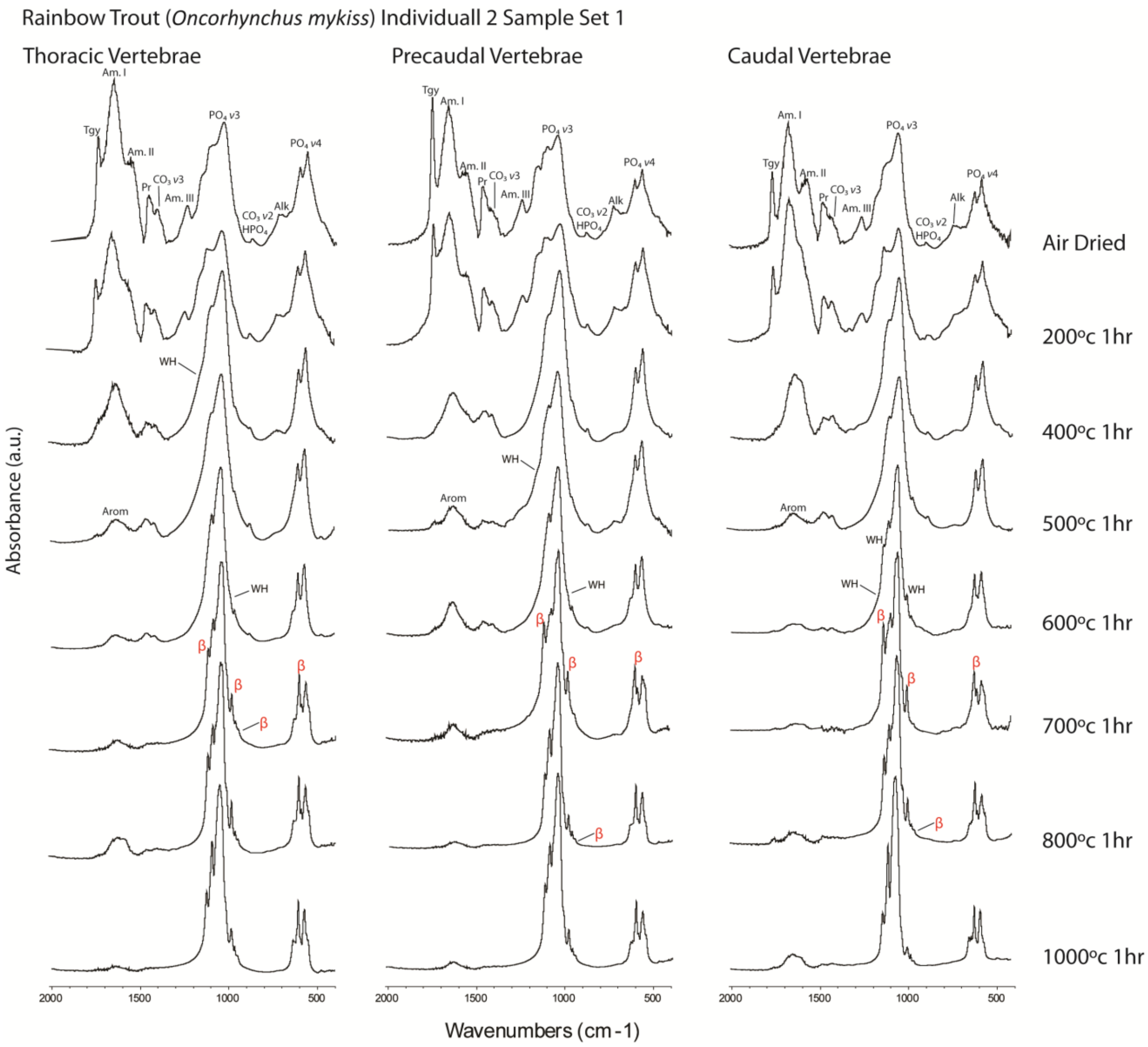

**Supplementary Figure 10: Rainbow Trout Individual 3 Vertebrae FTIR Replications (Fingerprint Region).** Abbreviations for diagnostic peaks: Tgy = triglyceride ester; Am = amide; Alk = alkene; Pr = proline; $CO_3$ = carbonate; $PO_4$ = phosphate; $HPO_4$ = hydrogen phosphate; Arom = aromatic char compounds; WH = whitlockite; β = beta magnesium tricalcium phosphate.

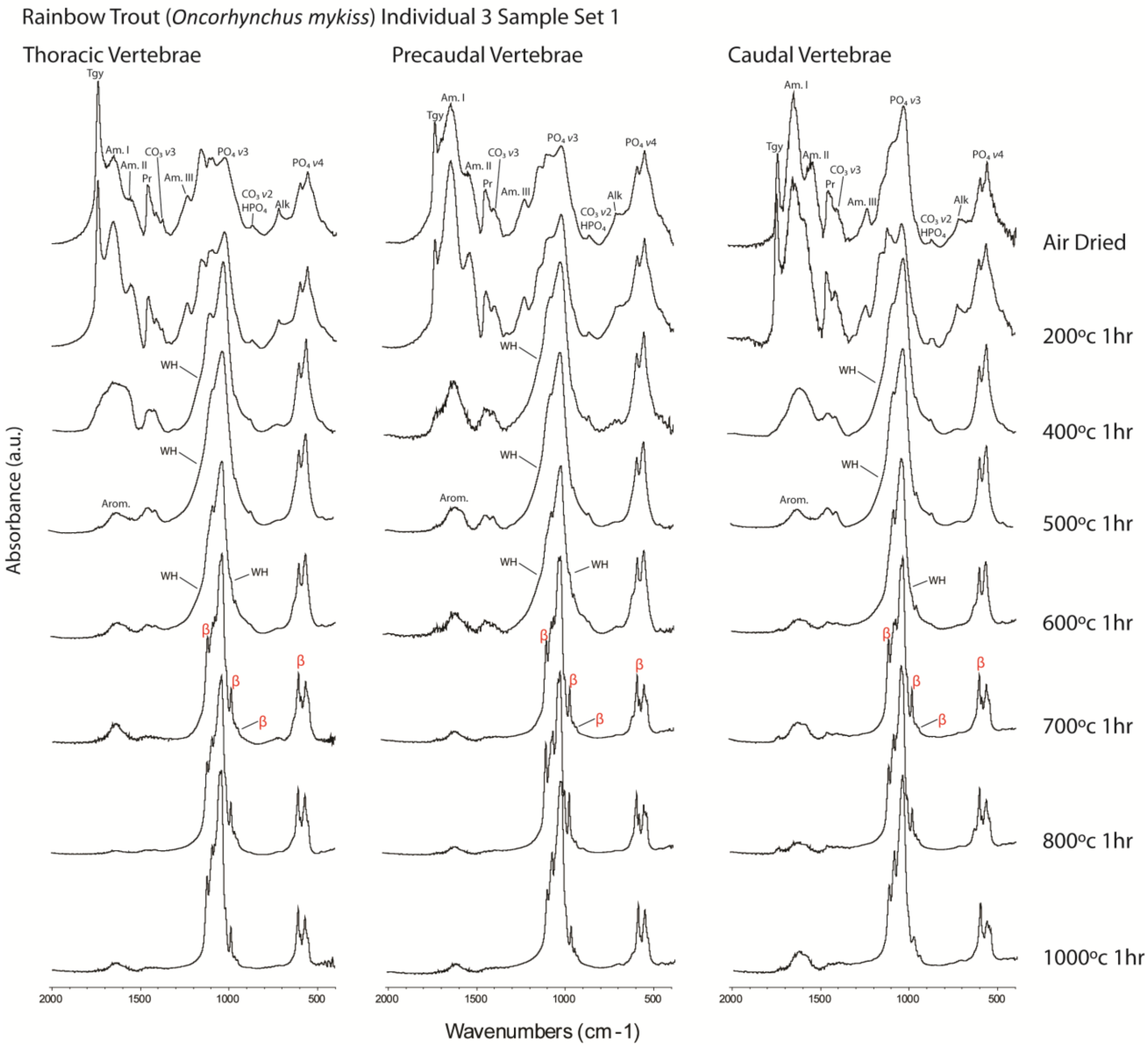

**Supplementary Figure 11: Atlantic Cod Individual 1 Vertebrae FTIR Replications (Fingerprint Region).** Abbreviations for diagnostic peaks: Tgy = triglyceride ester; Am = amide; Alk = alkene; Pr = proline; $CO_3$ = carbonate; $PO_4$ = phosphate; $HPO_4$ = hydrogen phosphate; Arom = aromatic char compounds; WH = whitlockite; β = beta magnesium tricalcium phosphate.

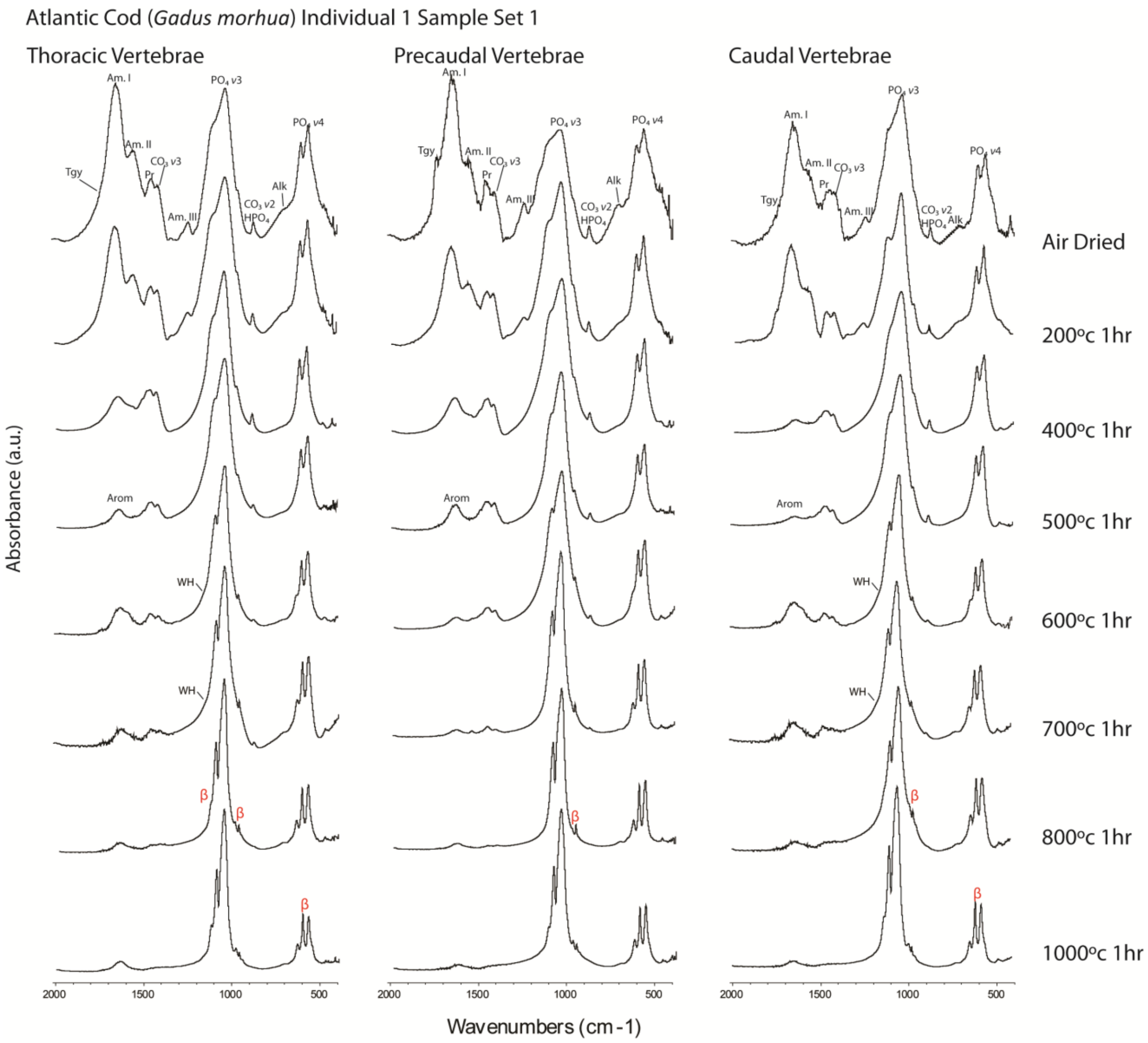

**Supplementary Figure 12: Atlantic Cod Individual 2 Vertebrae FTIR Replications (Fingerprint Region).** Abbreviations for diagnostic peaks: Tgy = triglyceride ester; Am = amide; Alk = alkene; Pr = proline; $CO_3$ = carbonate; $PO_4$ = phosphate; $HPO_4$ = hydrogen phosphate; Arom = aromatic char compounds; WH = whitlockite; β = beta magnesium tricalcium phosphate.

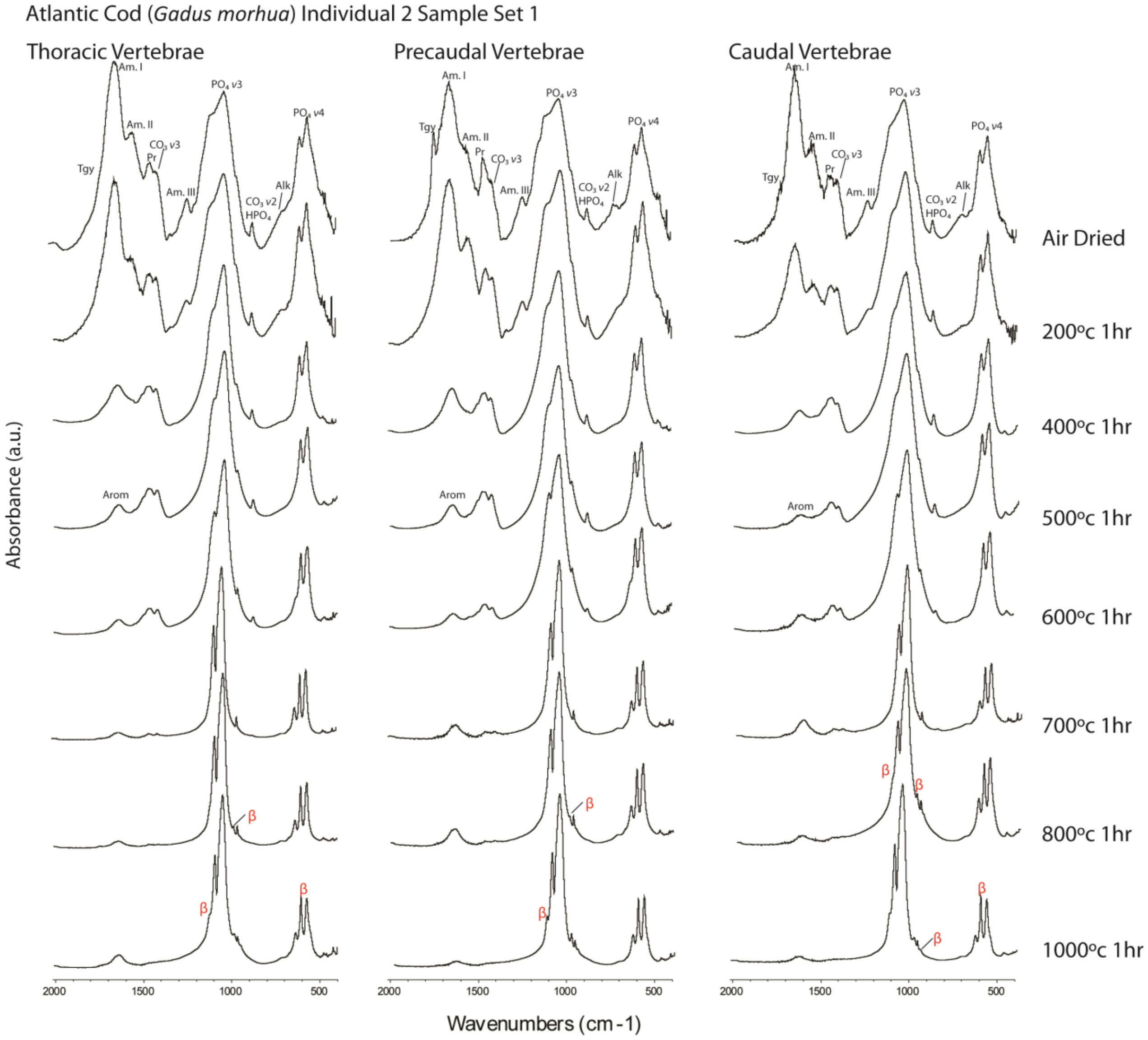

**Supplementary Figure 13: Atlantic Cod Individual 3 Vertebrae FTIR Replications (Fingerprint Region).** Abbreviations for diagnostic peaks: Tgy = triglyceride ester; Am = amide; Alk = alkene; Pr = proline; $CO_3$ = carbonate; $PO_4$ = phosphate; $HPO_4$ = hydrogen phosphate; Arom = aromatic char compounds; WH = whitlockite; β = beta magnesium tricalcium phosphate.

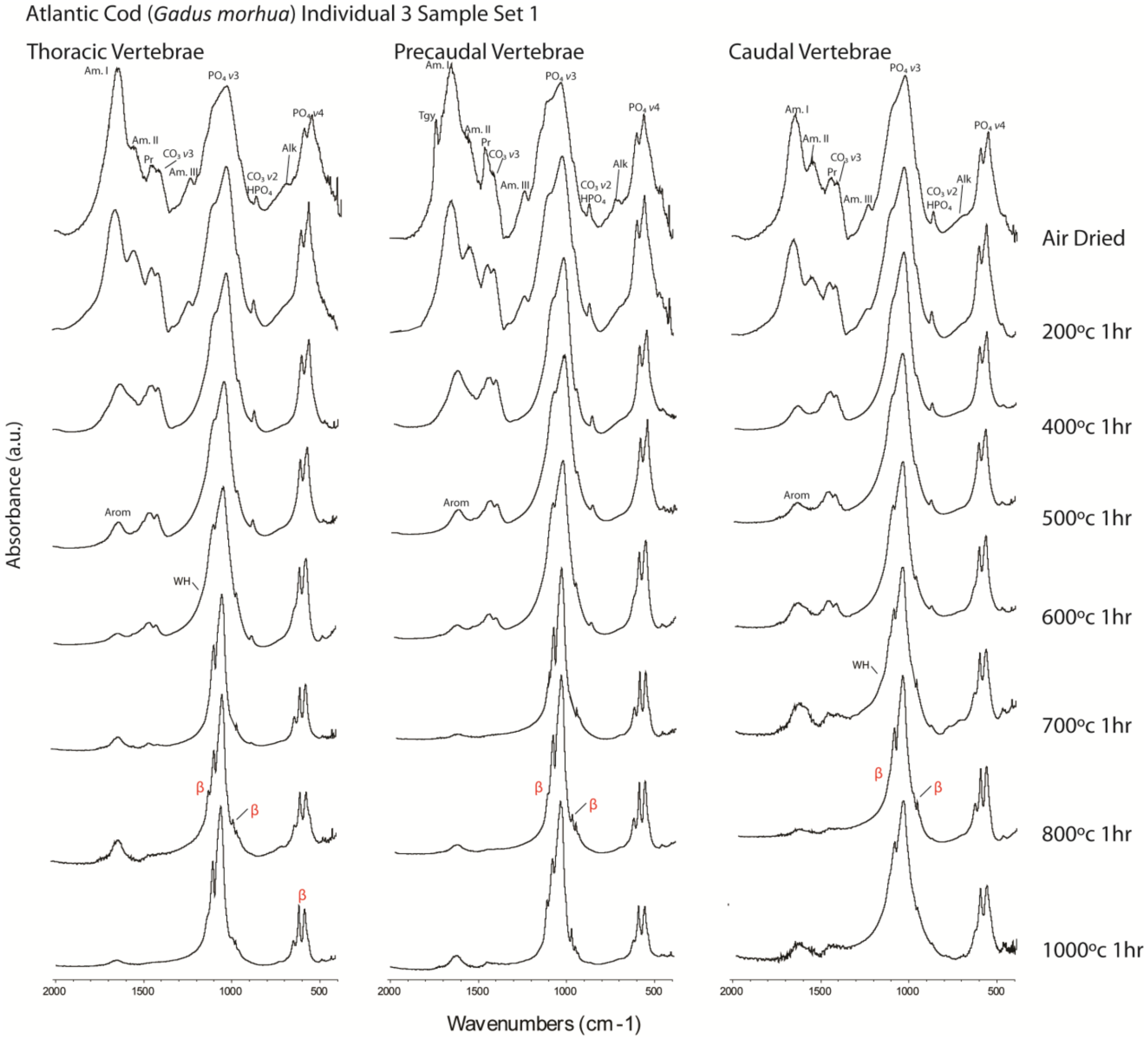

**Supplementary Figure 14: Caribou and Moose FTIR Replications (Fingerprint Region).** Abbreviations for diagnostic peaks: Tgy = triglyceride ester; Am = amide; Alk = alkene; Pr = proline; $CO_3$ = carbonate; $PO_4$ = phosphate; $HPO_4$ = hydrogen phosphate Arom = aromatic char compounds.

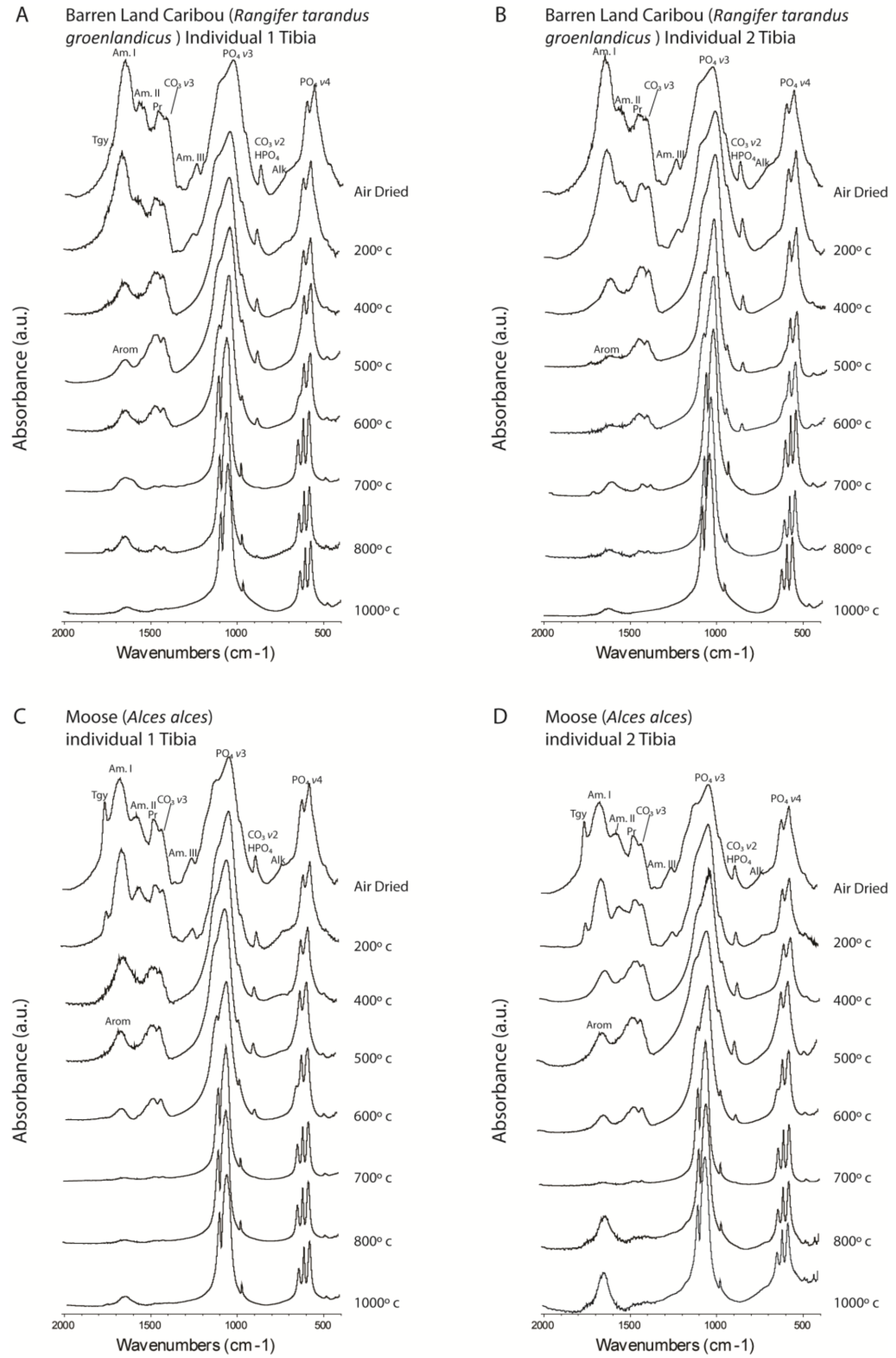

**Supplementary Figure 15: Duck FTIR Replications (Fingerprint Region).** Abbreviations for diagnostic peaks: Tgy = triglyceride ester; Am = amide; Alk = alkene; Pr = proline; $CO_3$ = carbonate; $PO_4$ = phosphate; $HPO_4$ = hydrogen phosphate; Arom = aromatic char compounds; β = beta magnesium tricalcium phosphate.

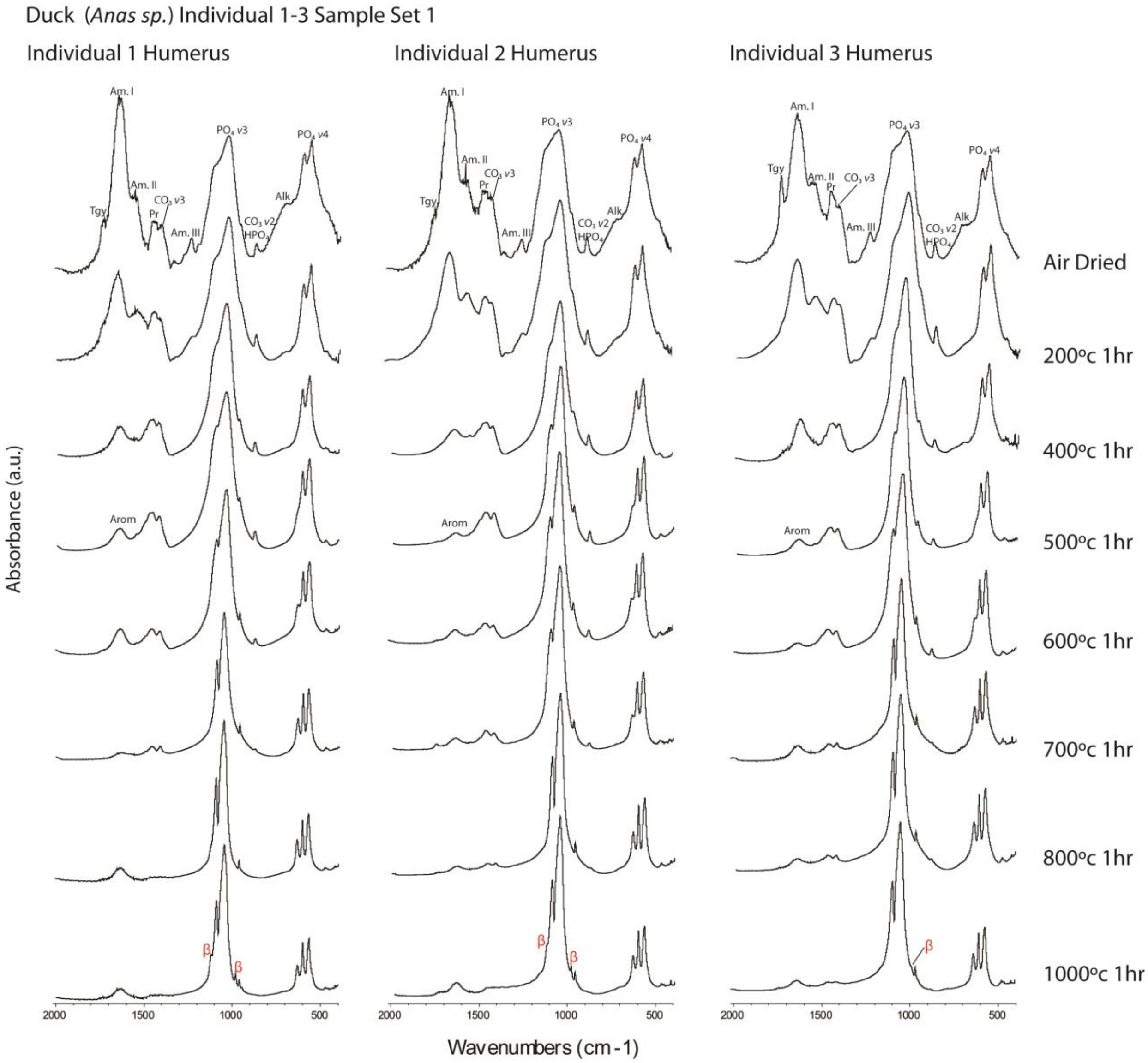

**Supplementary Figure 16: Ptarmigan FTIR Spectra (Fingerprint Region).** Abbreviations for diagnostic peaks: Tgy = triglyceride ester; Am = amide; Alk = alkene; Pr = proline; $CO_3$ = carbonate; $PO_4$ = phosphate; $HPO_4$ = hydrogen phosphate; Arom = aromatic char compounds.

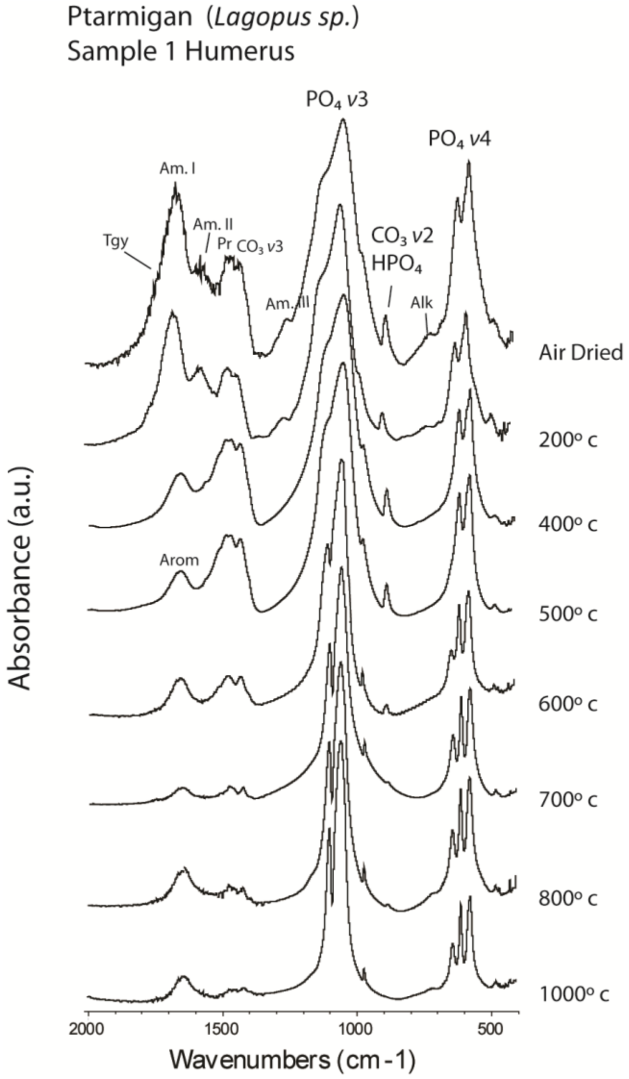

**Supplementary Figure 17: Representative FTIR Spectra for Salmon and Steelhead Vertebrae Showing Changes in the Phosphate *v*4 Absorbance Band** (HAp = hydroxylapatite; WH = whitlockite; β = beta magnesium tricalcium phosphate; OH = hydroxyl group).

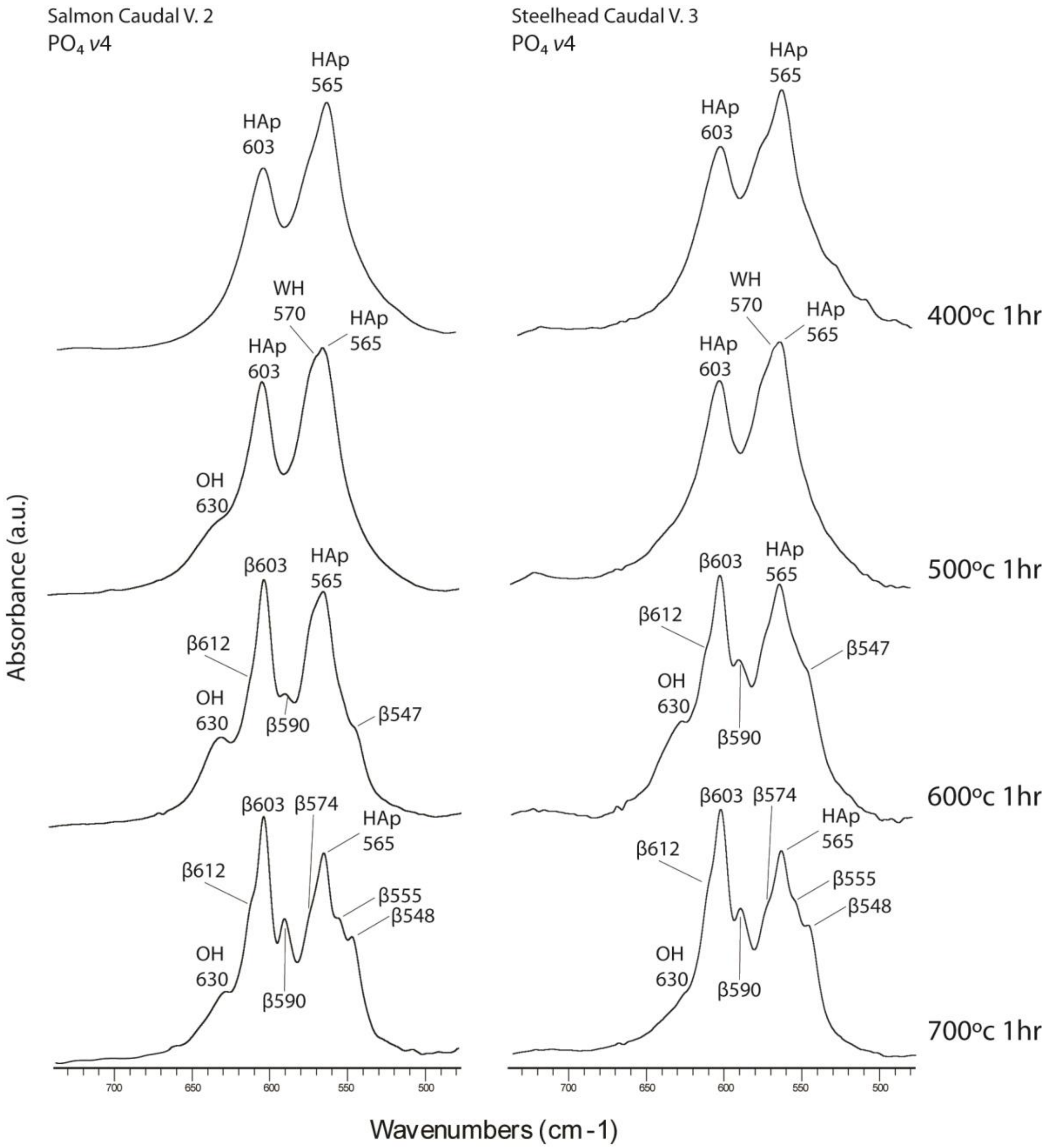

**Supplementary Figure 18: Observed XRD Diffractograms and Rietveld Refinements for Heat Treated Steelhead Bone.** Each inset displays the observed diffractogram on top followed by refinement results on bottom; diagnostic reflectance planes and corresponding 2 ϴ angles for each phase are indicated in the observed diffractograms; separated phases are presented in the refinement sections; hydroxylapatite (HAp) is represented in black, whitlockite (WH) in blue, and beta magnesium tricalcium phosphate (βMgTCP) in red; (**A**) steelhead caudal vertebra heat treated at 500 °C demonstrating diagnostic reflectance peaks for WH and HAp; (**B**) steelhead caudal vertebra heat treated at 600 °C demonstrating an increase in WH; (**C**) well developed biphasic HAp / βMgTCP in a steelhead caudal vertebra after treatment at 800 °C.

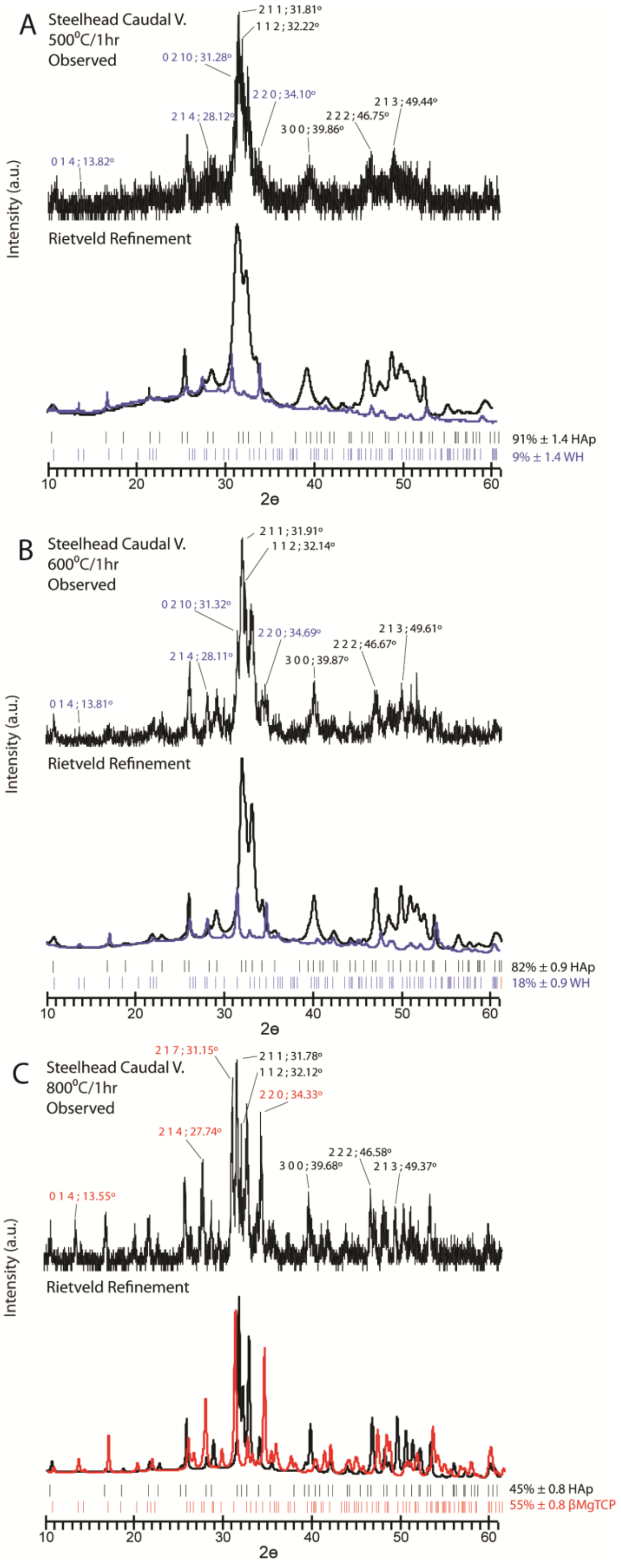

**Supplementary Figure 19: XRD Results for Phase Compositions Illustrating Variation in HAp-βMgTCP Ratios.** (**A**) Salmon caudal vertebra treated at 600 °C for 2 h; a 75:25% mineral ratio was produced; (**B**) Salmon caudal vertebra treated at 600 °C for 4 h; (**C**) Salmon caudal vertebra treated at 700 °C for 4 h; (D) Salmon caudal vertebra treated at 800 °C for 30 min.

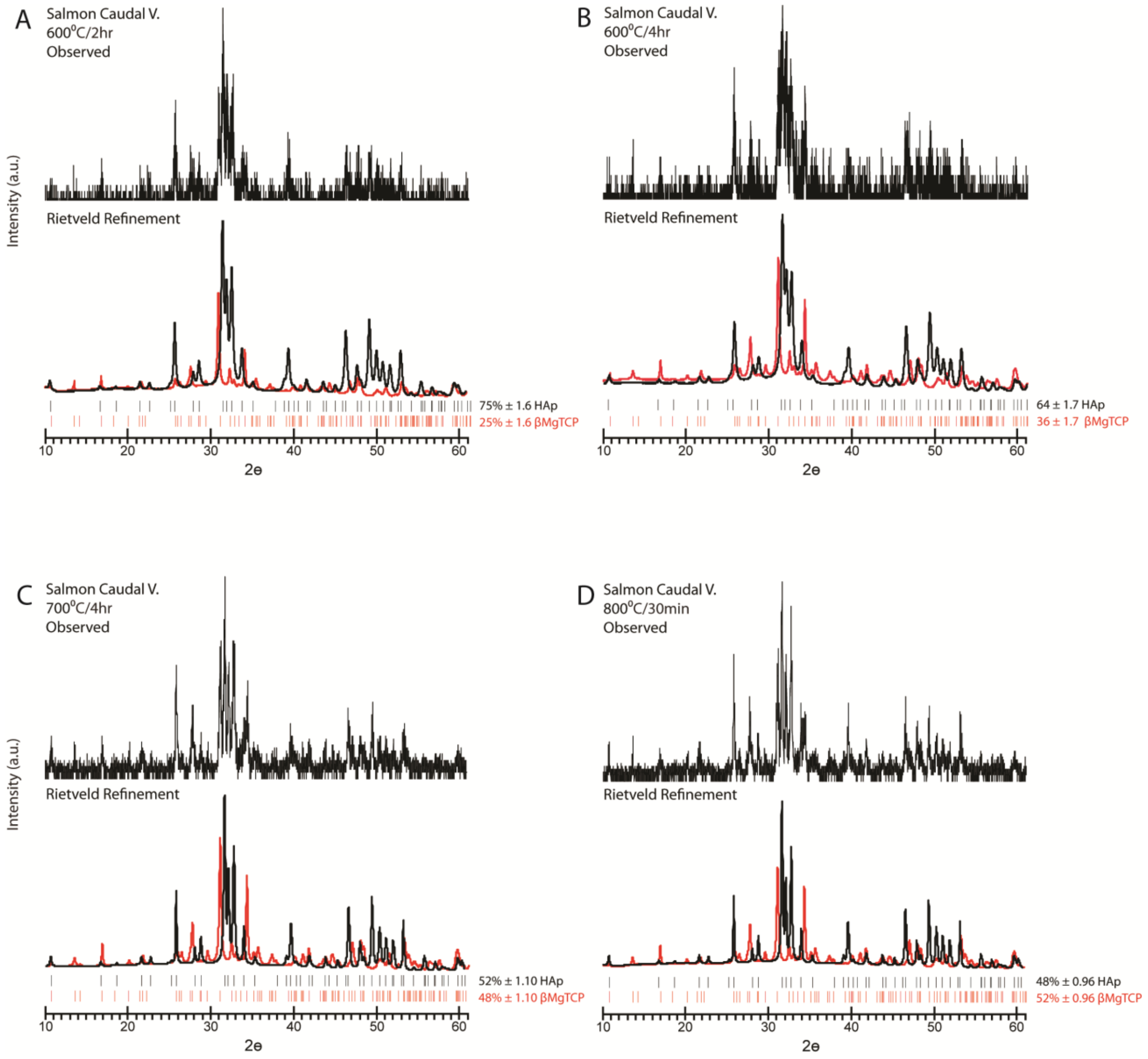

Supplement: Supplementary file 1 — Supplementary Figures [file 41598_2017_3737_MOESM1_ESM.pdf]
